# Remodeling of the Methylation Landscape in Breast Cancer Metastasis

**DOI:** 10.1371/journal.pone.0103896

**Published:** 2014-08-01

**Authors:** Marsha Reyngold, Sevin Turcan, Dilip Giri, Kasthuri Kannan, Logan A. Walsh, Agnes Viale, Marija Drobnjak, Linda T. Vahdat, William Lee, Timothy A. Chan

**Affiliations:** 1 Human Oncology and Pathogenesis Program, Memorial Sloan-Kettering Cancer Center, New York, New York, United States of America; 2 Department of Radiation Oncology, Memorial Sloan-Kettering Cancer Center, New York, New York, United States of America; 3 Department of Pathology, Memorial Sloan-Kettering Cancer Center, New York, New York, United States of America; 4 Genomics Core, Memorial Sloan-Kettering Cancer Center, New York, New York, United States of America; 5 Pathology Core, Memorial Sloan-Kettering Cancer Center, New York, New York, United States of America; 6 Department of Medicine, Weill Cornell Medical Center, New York, New York, United States of America; Northwestern University, United States of America

## Abstract

The development of breast cancer metastasis is accompanied by dynamic transcriptome changes and dramatic alterations in nuclear and chromatin structure. The basis of these changes is incompletely understood. The DNA methylome of primary breast cancers contribute to transcriptomic heterogeneity and different metastatic behavior. Therefore we sought to characterize methylome remodeling during regional metastasis. We profiled the DNA methylome and transcriptome of 44 matched primary breast tumors and regional metastases. Striking subtype-specific patterns of metastasis-associated methylome remodeling were observed, which reflected the molecular heterogeneity of breast cancers. These divergent changes occurred primarily in CpG island (CGI)-poor areas. Regions of methylome reorganization shared by the subtypes were also observed, and we were able to identify a metastasis-specific methylation signature that was present across the breast cancer subclasses. These alterations also occurred outside of CGIs and promoters, including sequences flanking CGIs and intergenic sequences. Integrated analysis of methylation and gene expression identified genes whose expression correlated with metastasis-specific methylation. Together, these findings significantly enhance our understanding of the epigenetic reorganization that occurs during regional breast cancer metastasis across the major breast cancer subtypes and reveal the nature of methylome remodeling during this process.

## Introduction

Breast cancer is a common malignancy that affected over 200,000 women in the US in 2012 and claimed nearly 40,000 lives [1]. It is a heterogeneous disease with several molecular subtypes defined on the basis of gene expression, initially described using immunohistopathological techniques and further refined on the basis of microarray profiling [2], [3]. Attesting to the clinical relevance of this classification, different subtypes are associated with distinct clinical outcomes [4], and subtype-specific therapeutic options have significantly impacted the natural course of this disease [5].

Despite significant progress, curative options are limited for metastatic disease that affects up to 40% of all women diagnosed with breast cancer. Presence of metastasis in the regional lymphatics is the most significant predictor of distant metastasis in breast cancer [6] and nearly always precedes it. Identifying the sequence of molecular events underlying metastatic spread will further our understanding of the metastatic process and contribute to therapy development efforts. To this end, several groups have analyzed expression profiles of primary tumors and regional or distant metastasis. Collectively these studies have shown that although at the transcriptome level, metastases are similar to the corresponding primary tumor, distinct differentially expressed genes in metastases converge on a number of common molecular pathways including extra-cellular matrix remodeling, adhesion, signal transduction and immune response [7]–[11]. However, these studies generally examined relatively few matched samples. In addition, recent studies examining the genomes of primary and metastatic lesions revealed few recurrent metastasis-specific mutations that could account for metastatic progression [12]–[15]. These findings support the hypothesis that although there are dynamic changes in cancer cells metastasizing from the primary site to lymph nodes or distant organs, the molecular underpinnings of this process remain poorly understood.

Cancer-specific DNA methylation changes are a hallmark of malignancies. Generally, gain of methylation on CpG-island associated promoters occurs in the context of global loss of methylation across the majority of the genome [16]. Promoter CpG-island hypermethylation has been shown to result in transcriptional silencing of many tumor suppressors. DNA hypomethylation can result in oncogene activation, and has been associated with loss of genomic integrity. We and others have recently shown that in primary breast tumors, global methylation patterns underlie many of the expression changes that define the molecular subgroups of breast cancer and metastatic risk [17]–[20]. However, the nature of epigenetic remodeling during the process of metastasis remains obscure.

To gain further insight into the molecular mechanisms of metastasis, and its initial steps, we investigated global methylome reprogramming and corresponding transcriptome changes in a large series of matched primary and regional metastases.

## Results and Discussion

### General Features of the Methylation Landscape from Primary Tumors are Maintained in Regional Metastases

**Figure 1:**
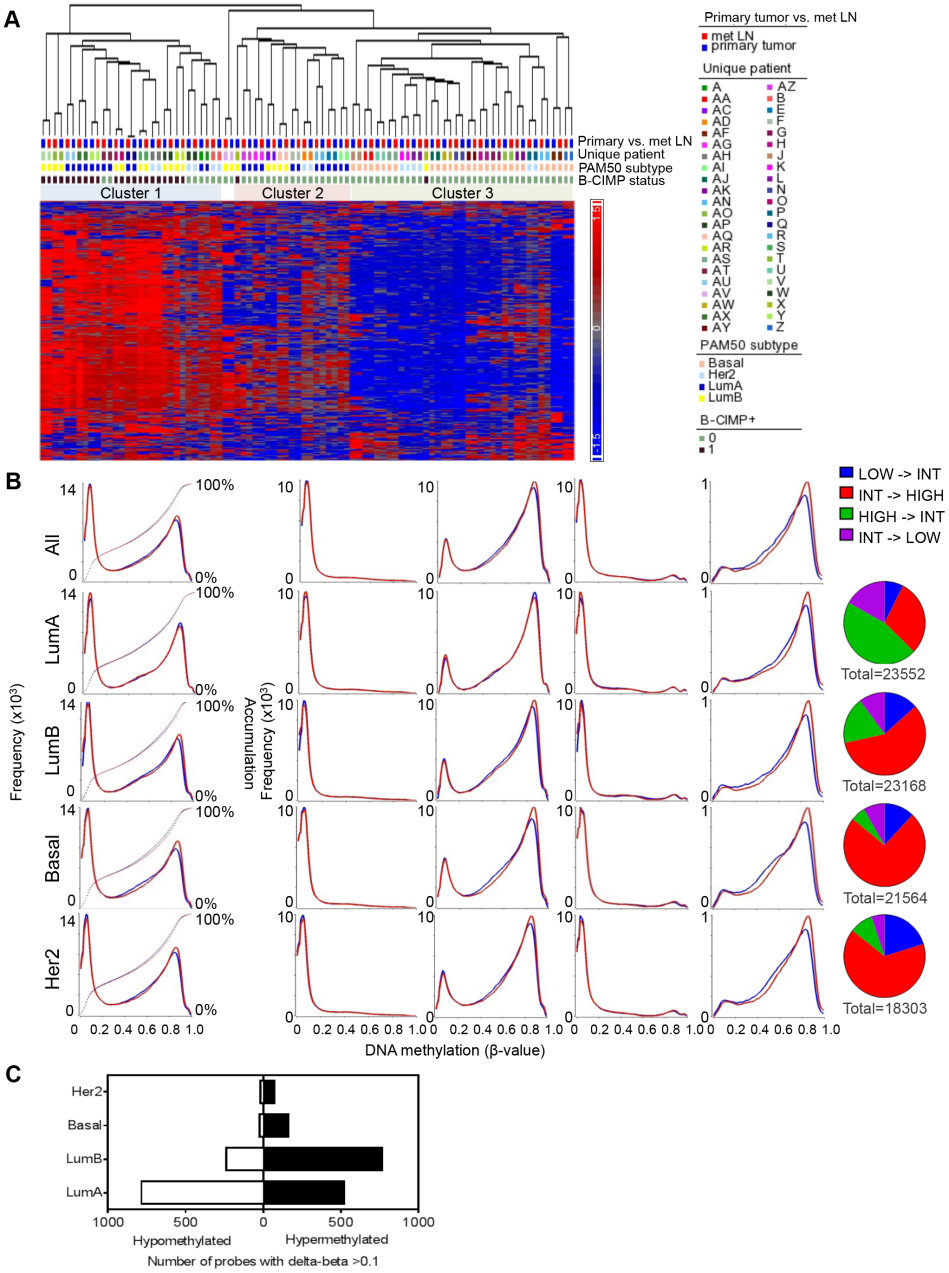
Methylation profiles of primary tumors are maintained in regional metastasis but display molecular subtype-specific differences. **A**. Dendogram shows unsupervised hierarchical clustering of methylation data from 44 matched primary breast carcinomas and regional metastases. A representative result using 4800 most variable probes is shown. Heat map displays relative methylation levels. Color scale indicates normalized β-value. Tumor characteristics are noted along the top of the heat map and labeled in the legend. **B**. Global methylation distributions for all probes (first column) and select functional subsets of probes. Data are shown for primaries and regional metastasis for all samples (top panel) and by molecular subgroup (lower panels). Y-axis, frequency of probes; X-axis CpG probe β-value. Quantification of all probes that underwent a change in methylation status from its initial category, including low (β-value ≤0.2), intermediate (β-value >0.2 and <0.7) and high (β-value ≥0.7) by subtype is shown on the far right. **C**. Quantification of probes that showed a change in methylation category with β-value difference of at least 0.10.

To characterize global changes in the methylome of breast cancers during regional metastasis, we determined DNA methylation profiles of 44 matched primary breast tumors and involved lymph nodes (Table S1) using the Illumina Infinium HumanMethylation 450 K array. Unsupervised hierarchical clustering of the most variant probes (n = 4,800–25,000) revealed that nearly all (43 of 44–98%) regional metastases clustered together with the corresponding primary tumor (**Fig. 1A**), demonstrating that the overall methylation landscape of each metastasis is more similar to its primary than to another metastasis. This shows that, in general, despite the gain in metastatic phenotype, overall features of the DNA methylome remain conserved between primary tumor and matched metastasis.

Consistent with previous reports of distinct methylation subgroups among primary breast carcinomas described by our group and others, clustering of matched primary-metastasis pairs segregated into several clusters [1], [17], [19]. Division into two main branches was mainly dependent on B-CIMP status [1]. As previously shown, the B-CIMP+ cluster was dominated by hormone-receptor positive luminal tumors, with slight luminal B predominance. Further subdivision of the B-CIMP- tumors correlated with transcriptomic subgroups, with cluster 2 dominated by luminal A and cluster 3 containing almost exclusively basal-like tumors. Consistent with previously shown heterogeneous methylation and expression profiles of Her2-enriched tumors, Her2-enriched tumor-metastasis pairs, representing a small number of all samples in this study, were widely distributed and appeared within all the clusters.

In addition to distinct patterns of methylation, different transcriptomic subtypes of breast cancer were associated with distinct patterns of DNA copy number aberrations (CNA). Therefore, we examined focal breast cancer-specific CNAs between the primaries and corresponding regional metastases using data derived from the methylation arrays. The CNA profile of each metastasis closely mirrored that of the corresponding primary tumor and reflected previously reported mRNA subtype-specific changes including gain of 1q and loss of 16q in the luminal pairs and loss of 5q and gain of 10p in the basal-like pairs [18], [21]–[23] (**Fig. S1**). Further subclassification of breast primary-metastasis pairs using CNA profiling did not reveal correlations with the methylation clusters.

Together these data show that metastasis methylome landscape is dominated by patient-specific and molecular subtype-specific methylation patterns, rather than by metastasis-specific changes. This result mirrors several studies profiling gene expression between pairs of primary tumors and matched metastasis, and suggests that methylome remodeling underlying the ability of primary tumors to metastasize may be an early event in tumorigenesis. However, these findings do not rule out the presence of metastasis-specific events that build upon these methylation patterns occurring in the metastatic lesions.

### Subtype-Specific Methylome Remodeling in Regional Metastasis

In order to determine whether there were metastasis-specific alterations in the methylome, we compared global methylation levels between primaries and matched metastases. To this end, histograms showing the distribution of probes with different β-values were examined for each group. This analysis showed that metastases had a decrease in the number of loci with intermediate methylation level (β-value >0.2 and <0.7) and increase in the number of loci with high methylation level (β-value ≥0.7), consistent with global hypermethylation (**Fig. 1B**). This preferentially affected sequences that lie outside of core promoters and carry intermediate levels of methylation in the primary tumors.

It is unknown whether methylome remodeling is the same for all breast cancer subtypes or whether these patterns are different. Furthermore, genes underlying transcriptomic subtype specificity are often regulated by methylation [19]. This, together with the results of our clustering analysis, suggested that the metastasis methylation landscape may also change in a subtype-specific way. Therefore, we examined global methylation changes in each subtype separately. Interestingly, distinct patterns of global methylation remodeling were seen in different transcriptomic subtypes of breast cancer (**Fig. 1B** **, bottom 4 rows**). Compared to matched primaries, basal-like metastases showed the most significant gain of loci with high methylation level - which primarily targeted non-promoter regions - followed by the Her2 and luminal B subtypes. In sharp contrast, luminal A metastases showed a small increase in the number of loci with low methylation levels in regions within and outside of promoters, with simultaneous decrease in the number of loci with high methylation levels in regions outside of promoters, resulting in a net loss of methylation.

Intriguingly, CpGs located within CpG island (CGI) shores and shelves were consistently hypermethylated in metastasis of all subtypes. Sequences with lower CpG density such as CGI shores and shelves are overrepresented among tissue-specific and cancer-specific differentially methylated regions across a number of malignancies [24], [25]. In cancer, this is thought to reflect loss of sharply delineated methylation boundaries at CGIs [25], [26]. It appears that the processes at work to remodel CpG methylation at the shores and shelves in the primary tumor continue to operate during metastatic progression.

Next, we wanted to determine the degree of change in probes that underwent alterations in methylation. To address this question, we quantified the number of loci that underwent a change from a baseline methylation level - categorized as hypomethylated (β-value <0.2), intermediate (β-value ≥0.2 and <0.7) or hypermethylated (β-value ≥0.7). All subtypes showed similar frequency of alterations but with differing patterns (**Fig. 1B** **, right column**). Hypomethylation events were dominant in luminal A subtype, less frequent in luminal B, and represented a minority of altered loci in metastases from basal and Her2-enriched tumors. Moreover, when we restricted our analysis to methylation alterations with delta-beta value (Δβ) >0.1, much fewer loci were noted to be altered in basal and Her2-enriched metastases than in the luminal metastases. These results show that both the type and the extent of methylation change in metastases differs between tumors of different molecular classification.

**Figure 2:**
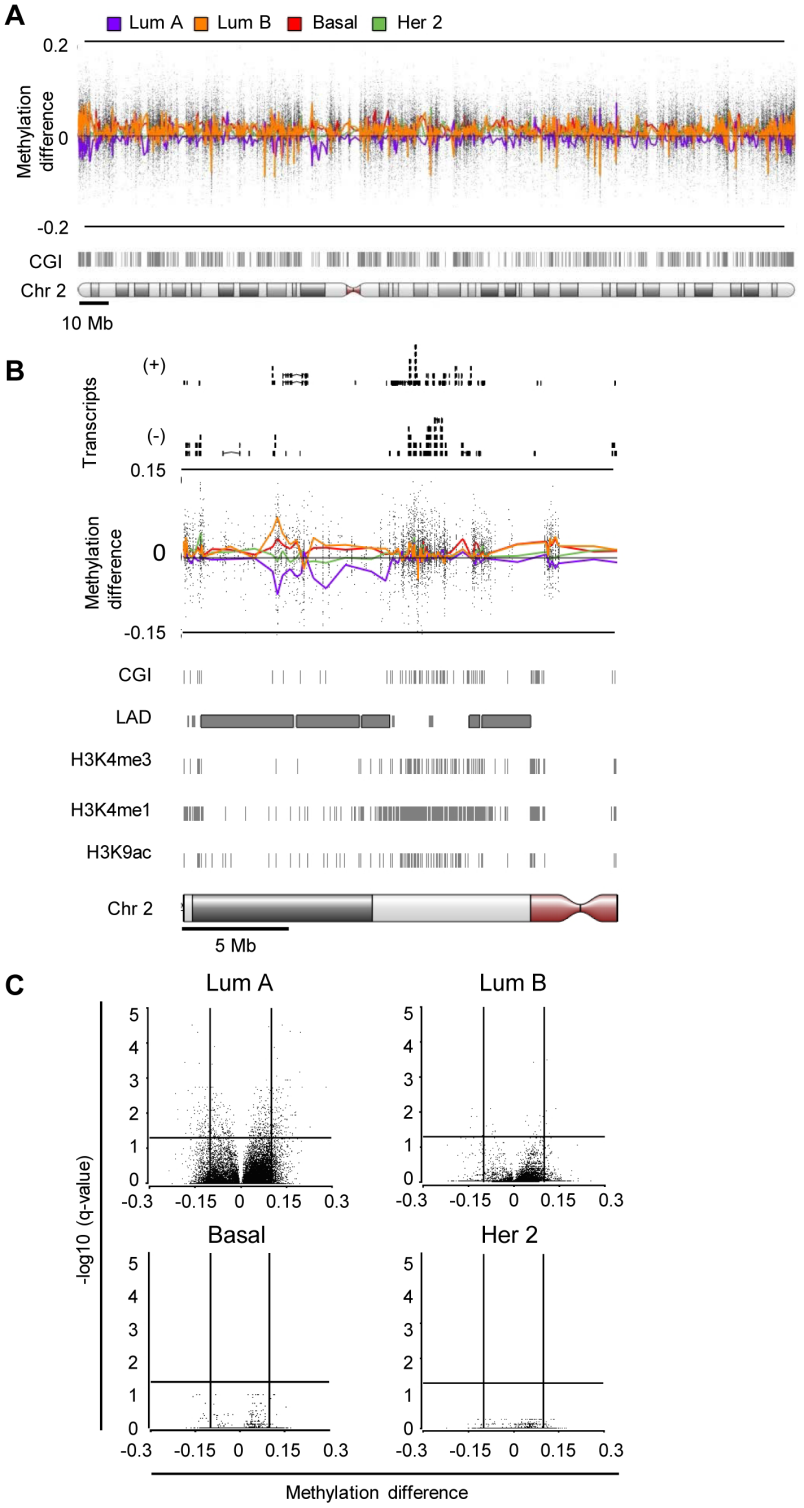
Chromosome characterization of subtype-specific methylation change in metastasis. **A**. Chromosome view of smoothed averaged paired differential methylation between metastasis and primary tumors shown for luminal A (purple), luminal B (orange), basal-like (red) and Her2-enriched (green) subtypes along human chromosome 2. CpG islands (CGI) are shown in grey below. **B**. Methylation profile of a 20 Mb region is shown. Location of RNAseq transcripts for+and – strands is shown above. CGIs, lamin B1-associated domains (LAD), and peaks for H3K4-trimethylated (H3K4me3), H3K4-monomethylated (H3K4me1) and H3K9-acetylated chromatin marks from human mammary endothelial cells (HMEC) are shown (from http://www.genome.ucsc.edu/cgi-bin/hgTables). **C**. Volcano plots of differentially methylated sites between metastasis and primaries by subtype-specific ANOVA. β-value difference is shown on the x-axis, -log10 of FDR-corrected p-value is on the y-axis. β-values of top three loci from luminal A, luminal B and basal primaries and metastases are shown in figure S1.

ANOVA was performed on each subtype to identify loci differentially methylated between the primaries and metastases of each group (**Fig. 2C**). In luminal A pairs, 614 probes were identified to be differentially methylated with FDR-corrected q-value of 0.05. In contrast, only 31 probes were identified in luminal B pairs, and 0 in both basal and Her2-enriched samples. Of note, this analysis is likely somewhat underpowered to identify small beta-value changes expected in basal-like and Her2-enriched samples on the basis of global profile analysis with statistical significance. However, given similar numbers of luminal A (n = 12), luminal B (n = 10) and basal-like (n = 15) pairs, our data illustrate that methylome remodeling is the most robust and concerted in the luminal subtypes, while methylome remodeling in basal and Her2 metastasis shows more variability (**Fig. 2C** and **Fig. S3**). In light of our prior findings that coordinate methylation change on a number of promoters (B-CIMP) is predominantly found in the ER-expressing primary tumors [17], the concerted methylome remodeling noted in luminal A, and to some extent luminal B metastasis may reflect evidence of the same process continuing to occur during metastatic progression.

Examination of methylation changes as a function of chromosomal location revealed that although DNA methylation variability occurred over the same general regions of chromosomes, differing patterns of methylation change were noted between the subtypes. Luminal A and B tumors showed the most significant methylation change from baseline at individual loci (**Fig. 2A–B**). Most notably, regions of hypomethylation in the luminal A subtype overlapped with regions that were often hypermethylated in the other subtypes. Interestingly, the areas which harbored the most methylation across all sybtypes anticorrelated with nuclear lamin binding areas and correlated positively with activating chromatin marks and active mRNA transcripts. Areas that had divergent methylation in metastases between subgroup, on the other hand, could occur in areas both rich and depleted for lamin binding regions. These aberrations were more prevalent over regions with a low CGI density suggesting, perhaps, that structural features of chromosomes may play a role in establishing this pattern of remodeling.

Hypermethylation of CGIs, hypomethylation of large blocks encompassing hypermethylated CGI and loss of sharply delineated methylation boundaries in the regions flanking CGI have all been described in cancer [25], [26]. Generally, these are thought to represent different, but interrelated aspects of methylation deregulation in the process of tumorigenesis [26]. We now show that specific patterns of methylome remodeling can be more dominant during metastatic progression of the same cancer: while CGI shore and shelf hypermethylation was consistent among the subtypes, hypomethylation was dominant in only luminal A metastases and often targeted regions that were hypermethylated in other subtypes. This raises an interesting question regarding the mechanisms of methylation remodeling seen in different subtypes. Our data may reflect the existence of distinct precursor cells for each type of tumor inclined to remodel the methylome differently during metastasis. Alternatively, it may be that different subtypes adopt inherently different nuclear/chromatin organization, which is reflected in broad DNA methylation patterns [25]–[28] during the metastatic process.

### Identification of Shared Metastasis-Specific DNA Methylation Changes across All Breast Cancer Subtypes

**Figure 3:**
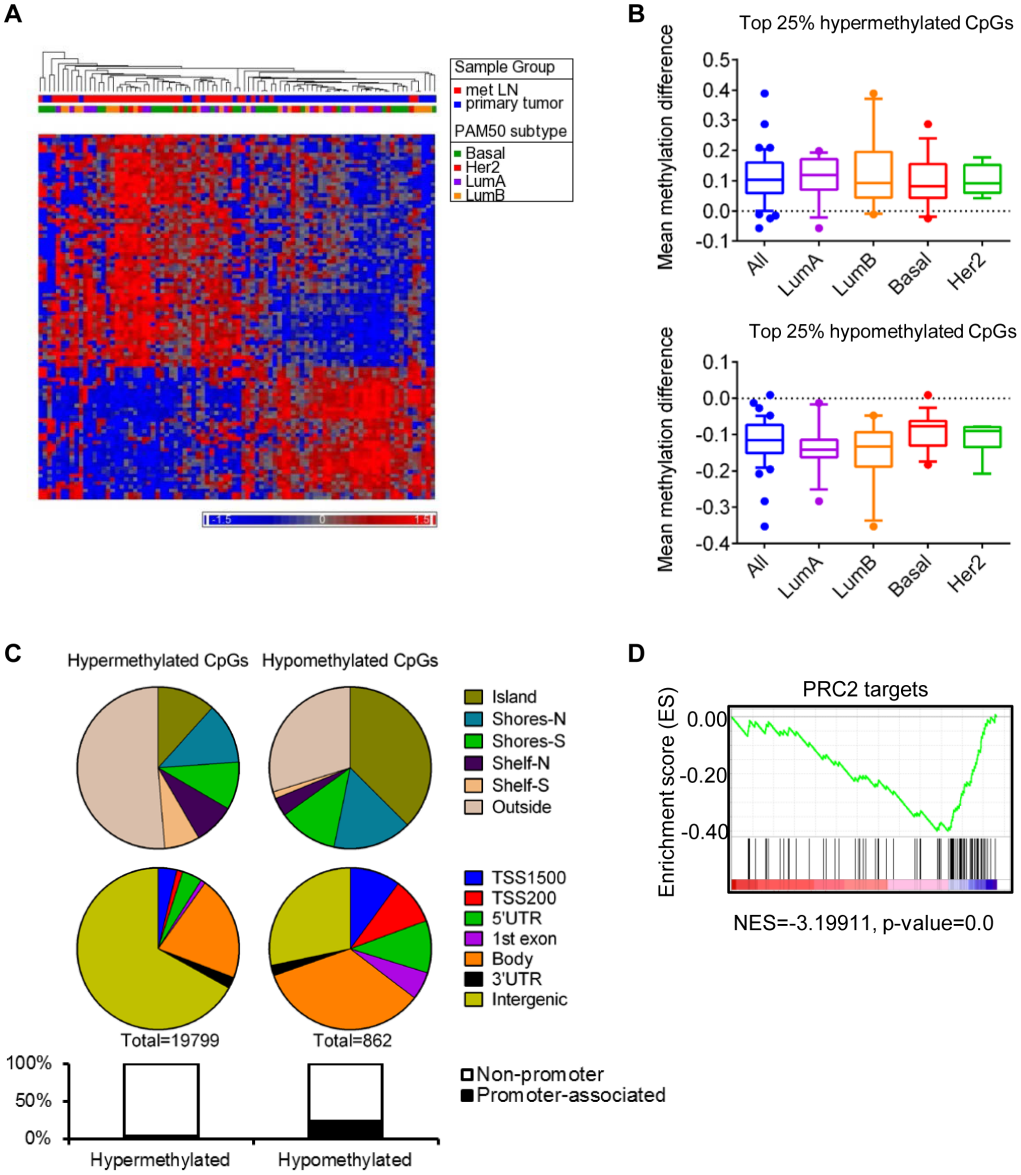
Methylome remodeling in metastasis across molecular subtypes of breast cancer. **A**. Hierarchical clustering of top 1000 differentially methylated loci (defined by SAM). Primary tumor or metastasis is noted along the top. PAM50 subtype classification is labeled. Color scale indicates normalized β-value. **B**. Box plots of metastasis-specific methylation change across top 25% differentially methylated probes common to all molecular subtypes. Y-axis, mean beta-value change. The median, 1 standard deviation (box plot), and 10–90 percentile (whiskers) are indicated in the graph. **C**. Frequency of differentially hypermethylated and hypomethylated loci as a function of relationship to CGIs (top panel), functional location (middle panel) and location within core promoter (bottom panel). **D**. Gene set enrichment analysis (GSEA) plot. GSEA was performed with PRC2 target list from Lee et al [37] The graph on the bottom represents the ranked, ordered, non-redundant list of genes (by SAM). Genes on the far left (red) correlated the most with metastases, and genes on the far right (blue) correlated the most primary samples. The vertical black lines indicate the position of each of the genes of the studied gene set in the ordered, non-redundant data set. The green curve corresponds to the ES (enrichment score) curve, which is the running sum of the weighted enrichment score obtained from GSEA software.

To identify differentially methylated regions between primaries and regional metastasis shared by all subtypes, paired significance analysis of microarrays (SAM) was performed. This analysis revealed that 19,799 CpGs were hypermethylated and 862 CpGs were hypomethylated (**Table S2**). Unsupervised hierarchical clustering revealed that separation of primary tumors from corresponding regional metastasis transcended transcriptomic subtypes (**Fig. 3A**). Consistent with distinct patterns of methylation change between molecular subtypes noted on global profiling, the amount of methylation difference at each shared metastasis-specific locus differed slightly across molecular subtypes (**Fig. 3B** and **Fig. S3**). The median methylation difference on the shared most differentially methylated loci was highest in the luminal A metastases, but the greatest variability in β-value change was seen in luminal B. Pairwise comparison of primary and regional metastasis β-values on several individual loci confirmed that the extent and variability of change on each site differed among the subtypes (**Fig. S2**).

Forty-nine percent of hypermethylated CpGs and 70% of hypomethylated CpGs were either within or were adjacent to a CGI (**Fig. 3C**). Notably, the majority of the hypermethylated CpGs related to a CGI (76%), and about half of the hypomethylation events (46%) occurred within the shores (0–2 kb from CGI) and shelves (2–4 kb from CGI). This is consistent with recent data that cancer-associated methylation change within CGI adjacent regions is greater than within the CGIs themselves [24].

We further analyzed functional location of differentially methylated loci. The majority of hypermethylated CpGs localized to intergenic regions. Consequently, only a small percentage of all hypermethylated loci, but 30% of all gene-associated loci, localized to a defined promoter or a promoter region encompassing TSS1500, TS200, 5′UTR and 1^st^ exon of a gene. Seventy percent of gene-associated, hypermethylated loci localized to a gene body or 3′UTR. In contrast, the majority of hypomethylated loci were related to genes, with 23% located within a defined promoter sequence, 35% falling within a larger promoter region encompassing TSS1500-1^st^ exon, and 36% located within gene body or 3′UTR.

Regions methylated in primary cancers have been shown to be enriched for polycomb complex 2 (PRC2) targets across a number of cancers [17], [29]–[31]. Therefore, we asked whether our metastasis-specific methylation changes occurred near PRC2 targets. GSEA analysis using a set of genes targeted by Suz12 (PRC2 component) showed that the methylation changes during metastasis preferentially occurred in genes that were not polycomb related, which contrasts with the preferential hypermethylation of PRC2 targets in primary tumors (NES = −3.19911, nominal p-value = 0) (**Fig. 3D**).

Polycomb-mediated H3K27 methylation has been shown to target certain genes for aberrant CGI methylation in cancer [29], [31]. Moreover, PRC2 targets are enriched in the B-CIMP specific gene set suggesting that this process is active in certain subtypes of breast cancers [17]. These results suggest that the process of establishing cancer-specific methylation is well underway during formation of the primary tumor, and that progressive remodeling of the methylome during metastatic progression occurs mainly outside of CGIs and may progress in a subtype-specific manner. Interestingly, it has been suggested that PRC2 core subunits *EED* and *EZH2* are expressed at a higher level in metastasis vs. primary breast tumors [32] raising an interesting possibility of continued functional relationship between the DNA methylome and this chromatin regulating complex.

### Gene Expression Profiling of Primary Breast Tumors and Matched Regional Metastases

**Figure 4:**
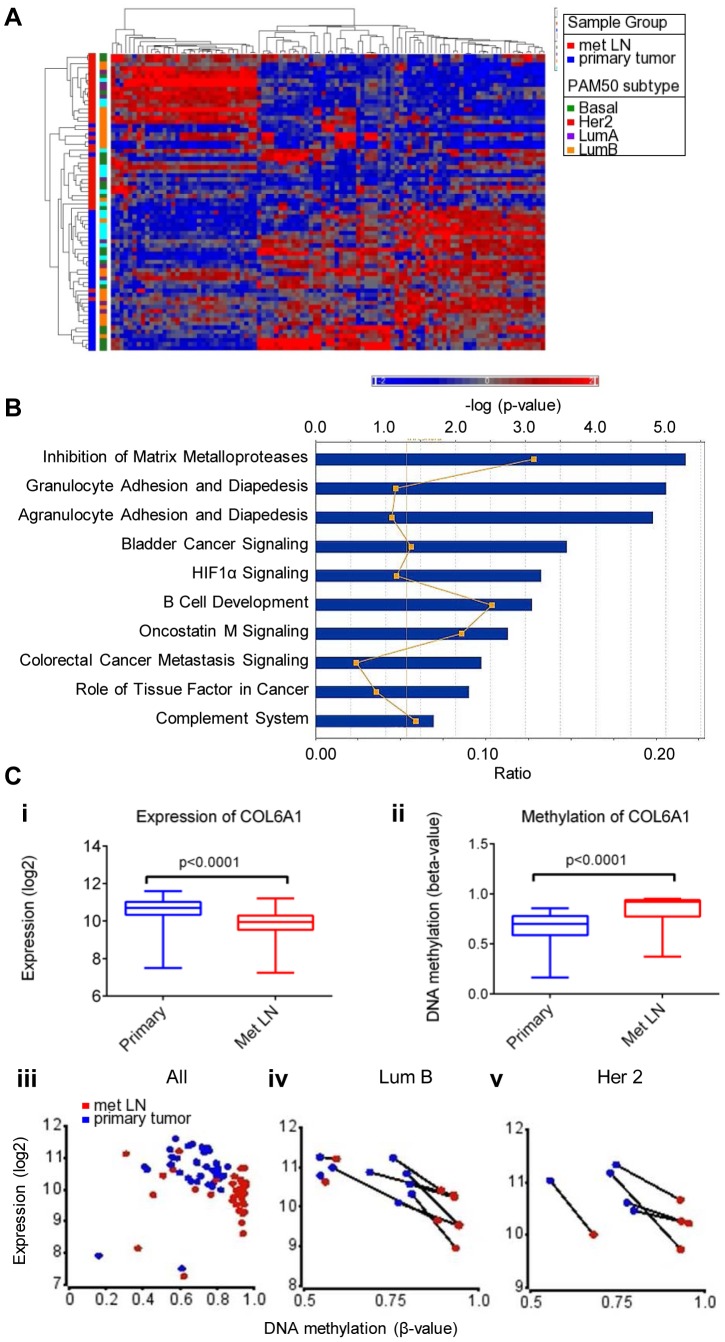
Integrated analysis of methylation and gene expression. **A**. 80-gene expression signature separates primary breast cancers from regional metastasis. **B**. Top canonical pathways associated with differentially methylated genes by Ingenuity IPA. The bar chart displays the identified pathways along with their significance (FDR-corrected Fisher’s exact test). Ratio of the number of genes from the dataset that map to the pathway divided by the total number of molecules in a given pathway that meet the criteria, divided by the total number of molecules that make up that pathway is displayed in orange. **C**. Hypermethylation of the promoter region of COL6A1 gene correlates with reduced expression in regional metastasis. Decrease in expression (i) and increase in methylation (ii) in metastases (red) as compared to primaries (blue) are shown. Correlation between expression and methylation for all pairs (iii) or in select molecular subtypes (Luminal B-enriched, iv) and (Her 2, v). Matched pairs are indicated by connecting lines.

To interrogate the transcriptional differences between primary tumor and matched metastases, we isolated RNA and analyzed it using Affymetrix 133A 2.0 microarrays. RNA from frozen primary tumor and matched regional metastasis were available for 36 patients of 44 pairs used for methylation profiling, including 12 with basal PAM50 subtype, 10 with luminal A subtype, 9 with luminal B subtype, and 5 with Her2 overexpression subtype. To identify differentially expressed genes, paired significance analysis of microarrays (SAM) was performed. 102 probes corresponding to 80 unique genes were differentially expressed, including 34 genes with higher and 67 genes with lower expression in the lymph nodes (**Fig. 4A** and **Table S3**). Among the top ranked genes were 35 genes (40%) that were previously identified to be differentially expressed in metastases. Downregulated genes included *ASPN, CCL8, COL11A1, CSTK, DIO2, FMO1, FST, GRP, ITGBL1, KRT14, LRRC15, MFAP5, MME, MMP1, MMP2, MMP3, MMP10, MMP13, MXRA5, OGN, PDGFRL, RPB4, SPOCK1, SPON1, and WNT2*. Upregulated genes included *C7, CCL21, CCDC102B, CD79B, CR2, EPHA3, FOXF1, GP1BA, MS4A1, TCL1A*. Notably, the gene expression signature was shared by all transcriptomic subgroups of breast cancer, suggesting that despite significant differences between subgroups there is a gene expression program that is common to all breast metastases (**Fig. 4A**).

Gene ontology classification on genes upregulated in the metastatic lymph nodes showed an association with pathways regulating hematopoietic cell lineage (p-value = 2.3×10^−5^) and B-cell receptor signaling (p-value = 4.7×10^−4^). This list was enriched for genes encoding transmembrane and plasma-membrane associated proteins and glycoproteins. The differentially downregulated set was highly enriched for genes encoding secreted proteins (1.9E-18), signaling molecules (1.4E-14), metalloproteinases (4.0E-9), extracellular matrix genes (1.2E-14), and genes involved in collagen degradation (9.4E-8). Likewise, pathway analysis using Ingenuity tool showed that gene set differentially expressed in metastases was enriched for canonical pathways related to extracellular matrix remodeling, inflammatory response and metastasis signaling (**Fig. 4B**).

### Integration of Metastasis-Specific Alterations Gene Expression and DNA Methylation

To determine the effects of DNA methylome changes on gene expression, we integrated expression and methylation data generated from our paired breast primaries and regional lymph nodes. Strikingly, analysis of differential methylation between primaries and matched regional metastases revealed that only 155 genes (121 hyper and 34 hypomethylated genes) showed methylation differences with a β-value of ≥0.15 in at least one molecular subtype of breast cancer. Integration of differentially expressed and methylated gene lists revealed that 8 genes showed significant change in methylation and expression (**Table S4**). One of the most consistently altered genes included the metastasis-associated gene *COL6A1* (**Fig. 4C**). In metastases, *COL6A1* became progressively more methylated than in matching primaries, and this was accompanied by a decrease in expression levels. These findings indicate that methylome remodeling in metastasis can provide additional changes in the metastatic lesions that directly affect gene expression, but this effect is limited in scope. This is in stark contrast to the major contribution of the methylome to shaping the transcriptomes of primary breast tumors, indicating that methylome remodeling associated with metastasis gene expression signatures is already present in the primary tumors and pointing to an early role for methylome remodeling in the tumorigenesis process [17], [18], [20].

In total, our data provide a detailed description of methylome remodeling in metastasis, showing that despite characteristic methylation patterns in different subtypes of breast tumors, there is concerted methylome reorganization in metastatic progression. Most remodeling identified in the metastatic lesions occurs outside of CGI and promoters, suggesting that promoter methylation is not the main mode of methylome alteration that occurs in metastasis. Methylation of CGIs and CGI-associated promoters constitute a small percentage of differentially methylated regions within metastasis, but may promote the metastatic phenotype. Large-scale metastasis-specific changes affect CGI poor areas and may reflect continuing reorganization of nuclear or chromatin structure during metastatic progression. These findings provide a framework for understanding the dynamics of methylome remodeling during metastasis and its impact on metastasis-associated expression programs. Our observations have substantial impact for advancing our understanding of epigenetic processes that affect metastatic progression.

## Materials and Methods

This study was approved by the IRB of Memorial Sloan-Kettering Cancer Center. All patients have given written informed consent.

Paired primary tumor and lymph node metastases (n = 44; Table S1) for methylation (n = 44) and gene expression analysis (n = 36) were collected at the time of primary surgery and banked at MSKCC. All samples were independently reviewed by a breast cancer pathologist and microdissected to obtain >70% tumor cell content. DNA and RNA were isolated using DNeasy Blood and Tissue and RNeasy Miniprep kits, respectively (QIAGEN). Methylation and gene expression profiling using Illumina Infinium 450 K methylation chip and Affymetrix GeneChip Human Genome U133 2.0 chip, respectively, were performed by the genome core in accordance with manufacturers’ instructions. Clustering was performed using Partek Genomics Suite (Partek). To identify differentially methylated probes in metastasis within each breast cancer subtype, we used analysis of variance (ANOVA). Paired significance analysis of microarrays (SAM) [33] was used to identify differentially methylated and expressed genes across all subtypes. Copy number aberrations were identified from the Infinium methylation array using the sum of methylated and unmethylated signal intensities based on a strategy described by Strum *et al* [34] using the circular binary segmentation algorithm developed by Venkatraman and Olshen [35] and Integrative Genomics Viewer to calculate and visualize the mean log-ratio of windows contained in each segment [36]. Additional experimental details are provided in *Materials and Methods S1.*

### Accession numbers

Methylation and expression datasets are deposited in the Gene Expression Omnibus under the accession number GSE59000.

## Supporting Information

Figure S1 IGV display of genomic (A–B) and gene-specific (C–D) CNAs in primary breast tumors and corresponding metastases. A. Summary of copy number gains (red) and losses (blue) are shown in the top panel. Heat maps of genome-wide CNAs in paired primary tumors and metastases are shown by subtype in the bottom panel. Each line corresponds to an individual specimen (primary tumor, red; metastasis, blue) for a single patient. Paired specimens for each patient are displayed consecutively. B. Genome-wide comparison of CNAs between metastases and corresponding primaries. Summary is shown in the top panel and subtype-specific heat maps are shown in the bottom panel. Each line represents a single patient. C. CNAs in select breast cancer-associated genes are shown for primary and metastasis pairs. D. Comparison of gene-specific CNAs between the metastasis and primary tumor for each patient.
(PDF)

Figure S2 Heterogeneity of methylome remodeling among subtypes. β-values for primaries and metastases are shown for 3 top differentially methylated probes for luminal A (A), luminal B (B) and basal-like (C) pairs by subtype-specific ANOVA.
(PDF)

Figure S3 Heterogeneity of methylation change on shared metastasis-specific differentially methylated CpGs by molecular subtype. β-values from primary tumors (blue) and metastases (red) are shown for select probes.
(PDF)

Table S1 Patient and sample characteristics. IDC, invasive ductal carcinoma; ILC, invasive lobular carcinoma; NE, neuroendocrine. ^1^clinical staging.
(DOCX)

Table S2 List of hyper and hypomethylated loci by subtype of breast cancer. SAM, Significance Analysis of Microarrays.
(DOCX)

Table S3 List of differentially expressed genes.
(DOCX)

Table S4 List of differentially methylated and expressed genes.
(DOCX)

Materials and Methods S1 (DOC)
