# Supplementary material for: Remodeling of the Methylation Landscape in Breast Cancer Metastasis
Source: PLoS One. 2014 Aug 1;9(8):e103896. doi: 10.1371/journal.pone.0103896 (PMC4118917; doi:10.1371/journal.pone.0103896)
Supplement: Figure S1 — IGV display of genomic (A–B) and gene-specific (C–D) CNAs in primary breast tumors and corresponding metastases. A. Summary of copy number gains (red) and losses (blue) are shown in the top panel. Heat maps of genome-wide CNAs in paired primary tumors and metastases are shown by subtype in the bottom panel. Each line corresponds to an individual specimen (primary tumor, red; metastasis, blue) for a single patient. Paired specimens for each patient are displayed consecutively. B. Genome-wide comparison of CNAs between metastases and corresponding primaries. Summary is shown in the top panel and subtype-specific heat maps are shown in the bottom panel. Each line represents a single patient. C. CNAs in select breast cancer-associated genes are shown for primary and metastasis pairs. D. Comparison of gene-specific CNAs between the metastasis and primary tumor for each patient. (PDF) [file pone.0103896.s001.pdf]

Supplementary Figure 1.

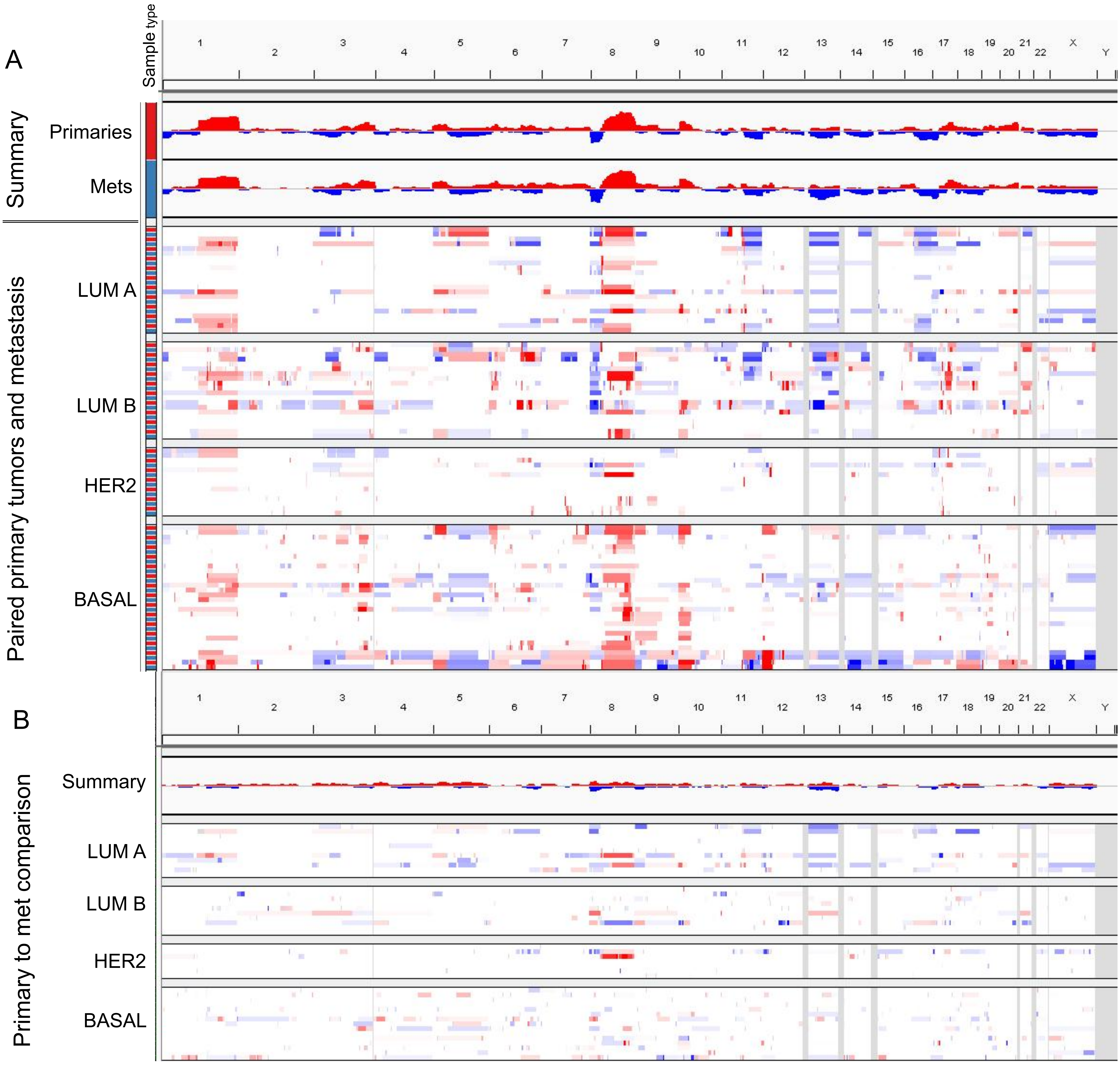

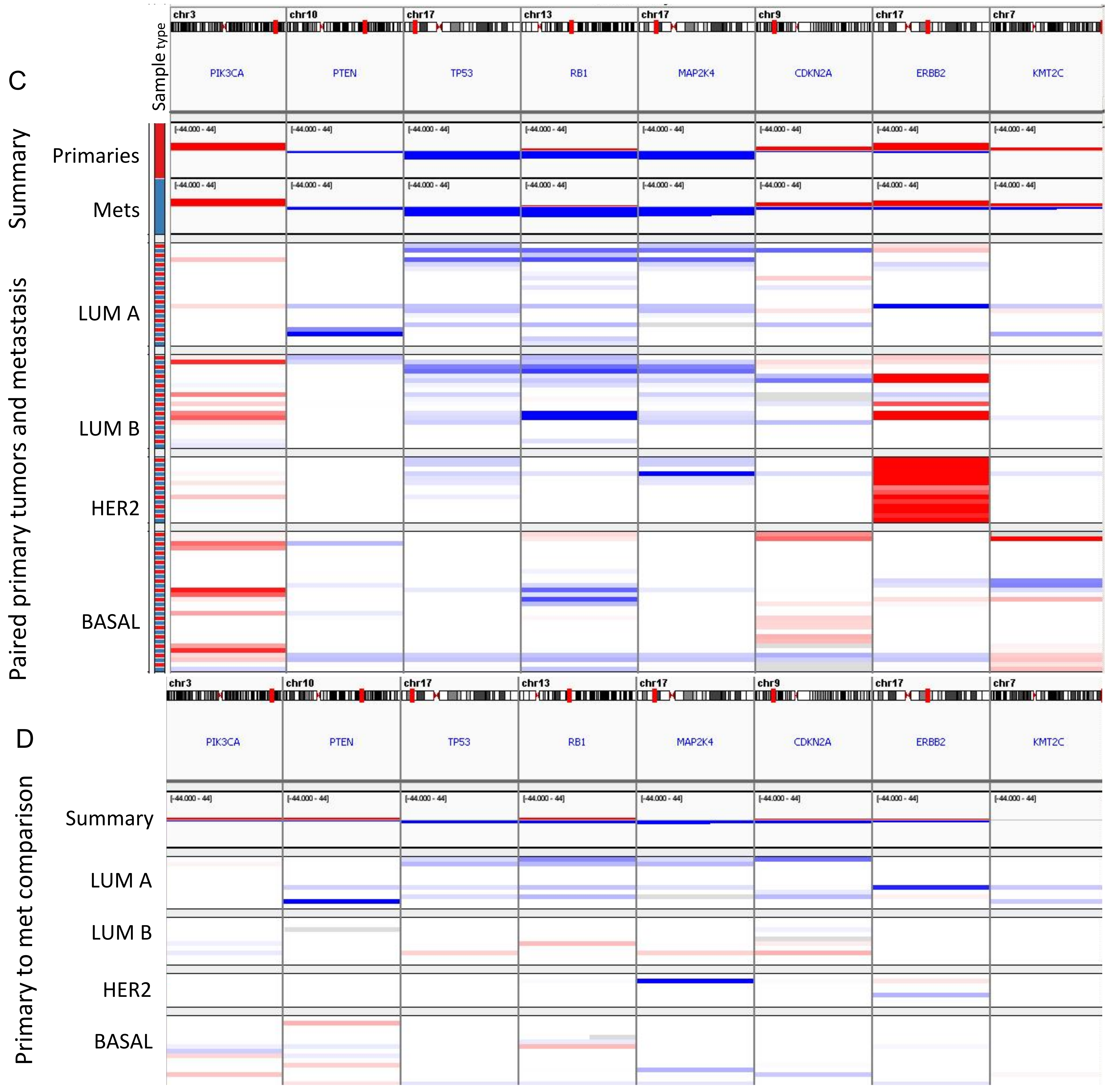

C
Sample type
chr3
chr10
chr17
chr13
chr17
chr9
chr17
chr7
PIK3CA
PTEN
TP53
RB1
MAP2K4
CDKN2A
ERBB2
KMT2C
Summary
Primaries
Mets
[-44.000 - 44]
Paired primary tumors and metastasis
LUM A
LUM B
HER2
BASAL
D
Primary to met comparison
Summary
LUM A
LUM B
HER2
BASAL
