# Supplementary material for: Remodeling of the Methylation Landscape in Breast Cancer Metastasis
Source: PLoS One. 2014 Aug 1;9(8):e103896. doi: 10.1371/journal.pone.0103896 (PMC4118917; doi:10.1371/journal.pone.0103896)
Supplement: Figure S2 — Heterogeneity of methylome remodeling among subtypes. β-values for primaries and metastases are shown for 3 top differentially methylated probes for luminal A (A), luminal B (B) and basal-like (C) pairs by subtype-specific ANOVA. (PDF) [file pone.0103896.s002.pdf]

Supplementary Figure 2.

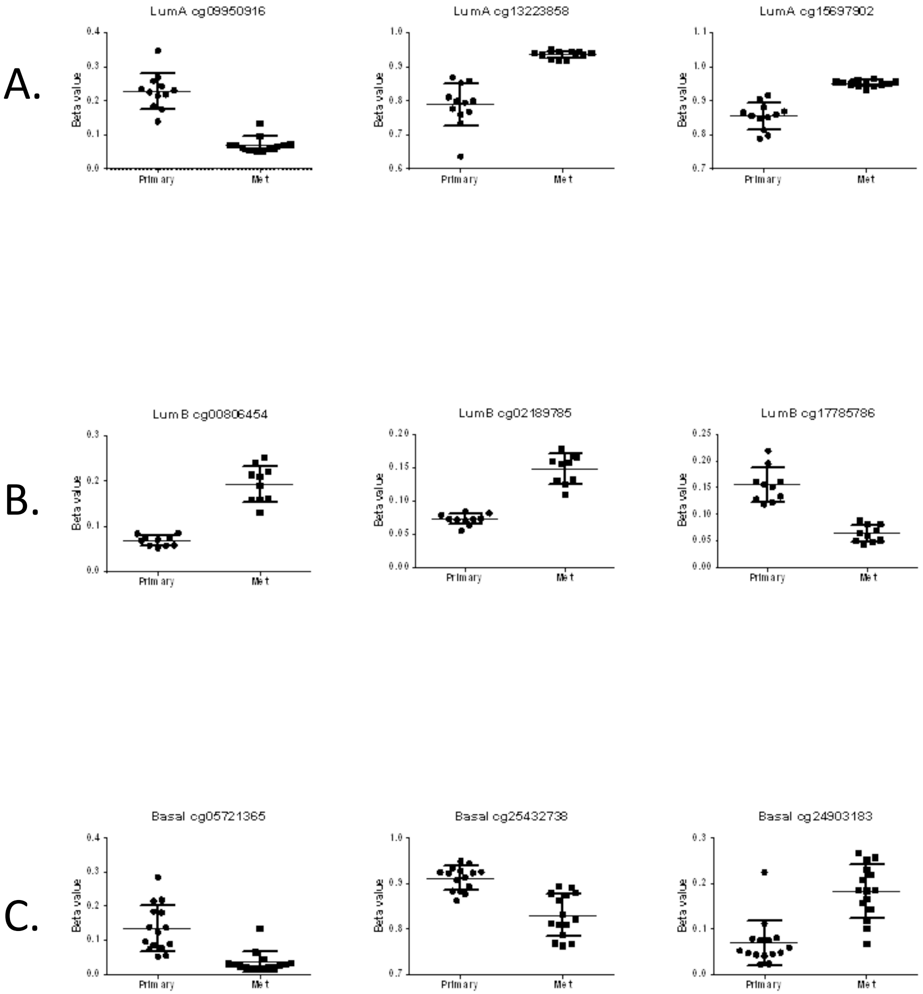

Figure S2. Heterogeneity of methylome remodeling among subtypes. β-values for primaries and metastases are shown for 3 top differentially methylated probes for luminal A (A), luminal B (B) and basal-like (C) pairs by subtype-specific ANOVA.
