# Supplementary material for: Remodeling of the Methylation Landscape in Breast Cancer Metastasis
Source: PLoS One. 2014 Aug 1;9(8):e103896. doi: 10.1371/journal.pone.0103896 (PMC4118917; doi:10.1371/journal.pone.0103896)
Supplement: Table S1 — Patient and sample characteristics. IDC, invasive ductal carcinoma; ILC, invasive lobular carcinoma; NE, neuroendocrine. 1clinical staging. (DOCX) [file pone.0103896.s004.docx]

Table S1. Patient and sample characteristics.

| Patient | Age at Diagnosis | Histology | AJCC 2010 Stage | T-stage | N-stage | M-stage | ER Status | PR Status | Her2 Final Status |
| --- | --- | --- | --- | --- | --- | --- | --- | --- | --- |
| Z | 45 | IDC | IIIA^1^ | T3^1^ | N2^1^ | M0 | Neg | Neg | Neg |
| Y | 68 | ILC | IIIC | T2 | N3 | M0 | Pos | Pos | Neg |
| X | 43 | IDC | IIIA | T3 | N1 | M0 | Pos | Pos | Neg |
| W | 45 | IDC | IIB | T2 | N1 | M0 | Pos | Pos | Neg |
| V | 63 | IDC | IIIC | T2 | N3 | M0 | Pos | Pos | Neg |
| U | 70 | IDC | IIB | T2 | N1 | M0 | Neg | Neg | Neg |
| T | 54 | invasive mammary carcinoma with NE features | IIB | T2 | N1 | M0 | Pos | Neg | Neg |
| S | 62 | IDC | IV | T2 | N3 | M1 | Pos | Pos | Neg |
| R | 66 | ILC+IDC | IIIA | T3 | N1 | M0 | Pos | Pos | Neg |
| Q | 50 | IDC | IIIA | T2 | N2 | M0 | Pos | Pos | Neg |
| P | 60 | IDC | IIIC | T2 | N3 | M0 | Neg | Neg | Pos |
| O | 47 | IDC | IV | T2^1^ | N1^1^ | M1 | Pos | Pos | Pos |
| N | 54 | IDC | IIB | T2 | N1 | M0 | Neg | Neg | Neg |
| L | 33 | IDC | IIA | T1 | N1 | M0 | Neg | Neg | Pos |
| K | 43 | IDC | IIIA | T1 | N2 | M0 | Neg | Neg | Pos |
| J | 43 | IDC | IIA^1^ | T1^1^ | N1^1^ | M0 | Neg | Neg | Neg |
| H | 54 | IDC | IIIC | T2 | N3 | M0 | Neg | Neg | Neg |
| G | 74 | IDC | IIIA | T3 | N1 | M0 | Neg | Neg | Neg |
| F | 45 | IDC+ILC | IIIC^1^ | T3^1^ | N3^1^ | M0 | Pos | Neg | Neg |
| E | 42 | IDC+ILC | IIIA | T3 | N2 | M0 | Pos | Pos | Neg |
| B | 49 | IDC | IIIA | T3 | N1 | M0 | Neg | Neg | Pos |
| AZ | 64 | IDC | IIA | T2 | N1 | M0 | Pos | Pos | Neg |
| AY | 35 | IDC | IIIC | T2 | N3 | M0 | Neg | Neg | Neg |
| AX | 61 | ILC | rIIIB^1^ | T4^1^ | N2^1^ | M0 | Pos | Pos | Neg |
| AW | 42 | IDC | IIB | T2 | N1 | M0 | Neg | Neg | Neg |
| AV | 76 | IDC | IIB | T2 | N1 | M0 | Pos | Pos | Pos |
| AU | 80 | IDC | IIIA | T1 | N2 | M0 | Pos | Pos | Neg |
| AT | 65 | ILC | IIIA | T2 | N2 | M0 | Pos | Pos | Neg |
| AS | 51 | IDC | IIA | T1 | N1 | M0 | Neg | Neg | Neg |
| AR | 64 | IDC | IIB | T2 | N1 | M0 | Pos | Pos | Neg |
| AQ | 46 | IDC | IIIC | T2 | N3 | M0 | Pos | Pos | Neg |
| AP | 49 | IDC | IIIA | T1 | N2 | M0 | Neg | Neg | Pos |
| AO | 38 | IDC | IIIA^1^ | T3^1^ | N1^1^ | M0 | Neg | Neg | Neg |
| AN | 39 | IDC | IIIC^1^ | T3^1^ | N3^1^ | M0 | Neg | Neg | Neg |
| AK | 83 | IDC | IIIA | T2 | N2 | M0 | Neg | Neg | Neg |
| AJ | 40 | IDC | IIB | T2 | N1 | M0 | Neg | Neg | Neg |
| AI | 54 | IDC | IIA | T1 | N1 | M0 | Pos | Pos | Neg |
| AH | 60 | IDC | IIIA | T2 | N2 | M0 | Pos | Neg | Pos |
| AG | 75 | IDC | IIB | T2 | N1 | M0 | Pos | Pos | Neg |
| AF | 63 | IDC | IV | T3^1^ | N2^1^ | M1 | Neg | Neg | Neg |
| AD | 46 | IDC | IIIC | T3 | N3 | M0 | Neg | Neg | Pos |
| AC | 65 | IDC | IIIA | T1 | N2 | M0 | Pos | Pos | Neg |
| AA | 62 | IDC | IIA | T1 | N1 | M0 | Neg | Neg | Neg |
| A | 61 | ILC | IIIA | T2 | N2 | M0 | Pos | Pos | Neg |

IDC, invasive ductal carcinoma; ILC, invasive lobular carcinoma; NE, neuroendocrine

^1^clinical staging
