# Supplementary material for: Remodeling of the Methylation Landscape in Breast Cancer Metastasis
Source: PLoS One. 2014 Aug 1;9(8):e103896. doi: 10.1371/journal.pone.0103896 (PMC4118917; doi:10.1371/journal.pone.0103896)
Supplement: Table S2 — List of hyper and hypomethylated loci by subtype of breast cancer. SAM, Significance Analysis of Microarrays. (DOCX) [file pone.0103896.s005.docx]

Table S2. List of hyper and hypomethylated loci by subtype of breast cancer.

| Gene Name | Probe ID | Change in beta-value, by subtype | | | | | | | | | SAM | | | |
| --- | --- | --- | --- | --- | --- | --- | --- | --- | --- | --- | --- | --- | --- | --- |
|  |  | ALL | | BASAL | | HER2 | LUM A | | LUM B | | Score (d) | Numerator (r) | Denominator (s+s0) | q-value |
| C22orf30 | cg05233946 | 0.205866 | 0.155224 | | 0.199083 | | | 0.27427 | | 0.204492 | 12.3919 | 0.205866 | 0.016613 | 0 |
| COL6A1 | cg11401293 | 0.162634 | 0.124995 | | 0.189071 | | | 0.198814 | | 0.157171 | 9.59908 | 0.162634 | 0.0169427 | 0 |
| ANXA2 | cg22365276 | 0.157843 | 0.13988 | | 0.127016 | | | 0.170531 | | 0.191142 | 5.33253 | 0.157843 | 0.0296001 | 0 |
| C1orf159 | cg19999567 | 0.156093 | 0.111681 | | 0.166374 | | | 0.231106 | | 0.1255 | 8.58856 | 0.156093 | 0.0181746 | 0 |
| LOC100130872-SPON2 | cg17227257 | 0.14168 | 0.0870075 | | 0.177832 | | | 0.191126 | | 0.139046 | 5.57571 | 0.14168 | 0.0254102 | 0 |
| SH3BP5 | cg08003402 | 0.135214 | 0.103514 | | 0.145063 | | | 0.169089 | | 0.135221 | 6.48462 | 0.135214 | 0.0208516 | 0 |
| MIR1180 | cg05896714 | 0.133514 | 0.114975 | | 0.160687 | | | 0.139111 | | 0.135585 | 6.73125 | 0.127458 | 0.0189353 | 0 |
| FAM20C | cg26440261 | 0.131913 | 0.117502 | | 0.112227 | | | 0.167124 | | 0.125056 | 6.7138 | 0.131913 | 0.0196481 | 0 |
| CAMKK1 | cg03179291 | 0.129157 | 0.104908 | | 0.123558 | | | 0.149694 | | 0.144807 | 5.61149 | 0.129157 | 0.0230166 | 0 |
| WWP2 | cg26736200 | 0.125895 | 0.107577 | | 0.0935074 | | | 0.166202 | | 0.127676 | 8.23736 | 0.125895 | 0.0152834 | 0 |
| C3orf64 | cg22620797 | 0.125782 | 0.0714337 | | 0.163685 | | | 0.178213 | | 0.117856 | 8.08665 | 0.125782 | 0.0155543 | 0 |
| LOC100130872 | cg21082272 | 0.12459 | 0.083063 | | 0.143744 | | | 0.168779 | | 0.120444 | 6.92993 | 0.12459 | 0.0179785 | 0 |
| DYNC1LI2 | cg00448868 | 0.12124 | 0.125421 | | 0.0447933 | | | 0.120451 | | 0.16943 | 4.8429 | 0.12124 | 0.0250347 | 0 |
| NFE2L2 | cg17178175 | 0.118121 | 0.121549 | | 0.06647 | | | 0.0970119 | | 0.174465 | 4.32592 | 0.118121 | 0.0273054 | 0.00675692 |
| ADAM5P | cg14742937 | 0.117395 | 0.104023 | | 0.119912 | | | 0.179018 | | 0.0617456 | 7.95218 | 0.117395 | 0.0147627 | 0 |
| SLC35E2 | cg27391816 | 0.117305 | 0.127837 | | 0.0463849 | | | 0.110042 | | 0.159868 | 4.20848 | 0.117305 | 0.0278735 | 0.00675692 |
| ERGIC1 | cg10462778 | 0.117164 | 0.125771 | | 0.0719319 | | | 0.0924482 | | 0.165575 | 4.84239 | 0.117164 | 0.0241955 | 0 |
| CSTA | cg18618429 | 0.11593 | 0.147544 | | 0.121593 | | | 0.0772763 | | 0.110931 | 5.63599 | 0.11593 | 0.0205696 | 0 |
| SPATC1 | cg10145449 | 0.115554 | 0.081256 | | 0.134332 | | | 0.178713 | | 0.0780646 | 7.04414 | 0.108943 | 0.0154658 | 0 |
| PFKFB3 | cg12235073 | 0.115413 | 0.119493 | | 0.0833564 | | | 0.0983352 | | 0.152226 | 4.50083 | 0.115413 | 0.0256427 | 0 |
| ANXA1 | cg13591783 | 0.1153 | 0.0939941 | | 0.10105 | | | 0.123363 | | 0.147559 | 6.75552 | 0.1153 | 0.0170676 | 0 |
| YPEL1 | cg15170942 | 0.115209 | 0.0805406 | | 0.137196 | | | 0.168414 | | 0.087975 | 10.375 | 0.115209 | 0.0111045 | 0 |
| FER1L5 | cg20312012 | 0.114236 | 0.0952333 | | 0.0752107 | | | 0.107487 | | 0.178156 | 4.14931 | 0.114236 | 0.0275313 | 0.00675692 |
| BRD4 | cg20078972 | 0.113981 | 0.100347 | | 0.0405341 | | | 0.120968 | | 0.177459 | 4.12747 | 0.113981 | 0.0276152 | 0.00675692 |
| FNDC3B | cg12972064 | 0.113671 | 0.107778 | | 0.0937969 | | | 0.0922179 | | 0.162166 | 4.37254 | 0.113063 | 0.0258575 | 0 |
| C10orf26 | cg15615645 | 0.113468 | 0.104817 | | 0.0446717 | | | 0.124762 | | 0.16105 | 5.76887 | 0.113468 | 0.019669 | 0 |
| IGSF9B | cg19907915 | 0.113353 | 0.0981616 | | 0.140102 | | | 0.174106 | | 0.0445116 | 5.44635 | 0.113353 | 0.0208126 | 0 |
| PPM1F | cg25898577 | 0.113237 | 0.106348 | | 0.107198 | | | 0.0776051 | | 0.170557 | 4.51795 | 0.113237 | 0.0250639 | 0 |
| STIM1 | cg14849140 | 0.11304 | 0.078939 | | 0.162604 | | | 0.130751 | | 0.108244 | 9.57008 | 0.105696 | 0.0110445 | 0 |
| CTBP1 | cg12525219 | 0.11263 | 0.0792931 | | 0.102017 | | | 0.172858 | | 0.0977911 | 7.53273 | 0.11263 | 0.0149521 | 0 |
| HOXB3 | cg15255390 | 0.111836 | 0.115634 | | 0.166437 | | | 0.0509687 | | 0.140959 | 5.13458 | 0.111836 | 0.0217809 | 0 |
| CDT1 | cg26841114 | 0.111775 | 0.0702755 | | 0.0955461 | | | 0.202499 | | 0.0765155 | 6.78722 | 0.111775 | 0.0164685 | 0 |
| RAP1GAP | cg03727673 | 0.11119 | 0.0668341 | | 0.122521 | | | 0.147158 | | 0.126631 | 9.19009 | 0.11119 | 0.0120989 | 0 |
| MIR572 | cg04203702 | 0.110634 | 0.0859229 | | 0.135557 | | | 0.0681113 | | 0.181281 | 4.76231 | 0.110634 | 0.0232311 | 0 |
| IL18 | cg15418499 | 0.110575 | 0.110588 | | 0.0971359 | | | 0.0678005 | | 0.171294 | 4.65513 | 0.110575 | 0.0237535 | 0 |
| TCEA3 | cg01015663 | 0.10996 | 0.0902835 | | 0.070688 | | | 0.124539 | | 0.14947 | 5.85572 | 0.10996 | 0.0187782 | 0 |
| HTRA1 | cg06474225 | 0.109712 | 0.137099 | | 0.0778896 | | | 0.0373853 | | 0.177697 | 3.88397 | 0.109712 | 0.0282473 | 0.0204183 |
| AKT1 | cg15912732 | 0.109407 | 0.0789177 | | 0.114146 | | | 0.168776 | | 0.0805811 | 7.47066 | 0.109407 | 0.0146449 | 0 |
| C11orf53 | cg03554573 | 0.109379 | 0.0876825 | | 0.122449 | | | 0.213209 | | 0.0081791 | 4.60479 | 0.110406 | 0.0239763 | 0 |
| CDCP1 | cg09220326 | 0.108673 | 0.0814444 | | 0.0915547 | | | 0.161716 | | 0.0978452 | 6.62483 | 0.108673 | 0.0164039 | 0 |
| RYR1 | cg02453828 | 0.107714 | 0.0734072 | | 0.104699 | | | 0.164874 | | 0.0926932 | 6.28024 | 0.107714 | 0.0171513 | 0 |
| DDAH2 | cg09632273 | 0.107104 | 0.125505 | | 0.185141 | | | 0.0581509 | | 0.0836181 | 3.80615 | 0.107104 | 0.0281396 | 0.0204183 |
| TOLLIP | cg03101580 | 0.106575 | 0.0789617 | | 0.107366 | | | 0.16393 | | 0.0786163 | 8.36093 | 0.106575 | 0.0127468 | 0 |
| TJP3 | cg13569431 | 0.106004 | 0.0744676 | | 0.116489 | | | 0.157498 | | 0.0841778 | 8.64161 | 0.106004 | 0.0122667 | 0 |
| FGGY | cg19773937 | 0.105034 | 0.107465 | | 0.100955 | | | 0.0692379 | | 0.1472 | 4.38956 | 0.105034 | 0.0239282 | 0 |
| NFIC | cg03242964 | 0.104908 | 0.0457231 | | 0.138383 | | | 0.177928 | | 0.0826296 | 6.69599 | 0.104908 | 0.0156673 | 0 |
| KCNQ1 | cg07592519 | 0.104272 | 0.0850829 | | 0.183367 | | | 0.126543 | | 0.0509638 | 8.75037 | 0.104944 | 0.0119931 | 0 |
| GPR133 | cg13240326 | 0.104016 | 0.091192 | | 0.0984553 | | | 0.0848898 | | 0.150096 | 5.40063 | 0.104016 | 0.01926 | 0 |
| C2orf48 | cg23005797 | 0.103406 | 0.0632671 | | 0.0920784 | | | 0.155607 | | 0.108901 | 5.36615 | 0.103406 | 0.01927 | 0 |
| SRPX2 | cg05911774 | 0.102954 | 0.159932 | | 0.110964 | | | 0.0273478 | | 0.10261 | 3.95492 | 0.102954 | 0.026032 | 0.0204183 |
| KIAA1598 | cg08908131 | 0.102456 | 0.0839317 | | 0.0936256 | | | 0.0927955 | | 0.148014 | 6.8762 | 0.102456 | 0.0149 | 0 |
| SNX31 | cg26440289 | 0.102332 | 0.115832 | | 0.196229 | | | 0.0398465 | | 0.0913385 | 4.89977 | 0.102332 | 0.0208851 | 0 |
| CD9 | cg04057956 | 0.101424 | 0.0785399 | | 0.0668473 | | | 0.0909357 | | 0.172541 | 4.10351 | 0.101424 | 0.0247165 | 0.00675692 |
| INS-IGF2 | cg26337010 | 0.101092 | 0.0820461 | | 0.0914131 | | | 0.151451 | | 0.0760032 | 6.32535 | 0.101092 | 0.015982 | 0 |
| MYOF | cg26581982 | 0.10021 | 0.103641 | | 0.0706627 | | | 0.0485394 | | 0.177752 | 4.02369 | 0.10021 | 0.0249051 | 0.0204183 |
| CARKD | cg01636873 | 0.100169 | 0.0865651 | | 0.0274576 | | | 0.156444 | | 0.103945 | 7.45428 | 0.0993206 | 0.013324 | 0 |
| PRDM1 | cg19064302 | 0.0990125 | 0.100419 | | 0.064528 | | | 0.0723354 | | 0.153054 | 5.07345 | 0.0990125 | 0.0195158 | 0 |
| ZMIZ1 | cg04716021 | 0.098837 | 0.0600768 | | 0.0930174 | | | 0.173755 | | 0.0711488 | 6.21533 | 0.098837 | 0.0159021 | 0 |
| C19orf35 | cg21449569 | 0.0986782 | 0.0932292 | | 0.0865136 | | | 0.154143 | | 0.048809 | 7.18342 | 0.107073 | 0.0149055 | 0 |
| C3orf21 | cg06704969 | 0.0984864 | 0.0547641 | | 0.101288 | | | 0.168126 | | 0.0785413 | 7.89853 | 0.0984864 | 0.0124689 | 0 |
| FLII | cg06554744 | 0.0984805 | 0.113676 | | 0.0406013 | | | 0.152066 | | 0.0518992 | 5.88202 | 0.0984805 | 0.0167426 | 0 |
| LRIG1 | cg02012974 | 0.0981991 | 0.0978217 | | 0.0306511 | | | 0.0927603 | | 0.152575 | 3.79663 | 0.0981991 | 0.0258648 | 0.0204183 |
| PKD1L1 | cg15042302 | 0.0980348 | 0.0748937 | | 0.0439011 | | | 0.107892 | | 0.158811 | 4.92278 | 0.0980348 | 0.0199145 | 0 |
| HCCA2 | cg03784994 | 0.0972766 | 0.0676761 | | 0.0635927 | | | 0.158998 | | 0.0911903 | 8.92026 | 0.0884439 | 0.00991495 | 0 |
| OSBPL5 | cg06849723 | 0.0969705 | 0.10024 | | 0.0481606 | | | 0.0723715 | | 0.155751 | 4.33423 | 0.0969705 | 0.0223732 | 0 |
| KDELR3 | cg01640635 | 0.0968079 | 0.0651911 | | 0.0856923 | | | 0.156313 | | 0.0806079 | 6.67512 | 0.0968079 | 0.0145028 | 0 |
| SDK2 | cg06061257 | 0.0966098 | 0.0707905 | | 0.0797689 | | | 0.156391 | | 0.07539 | 5.08236 | 0.0966098 | 0.0190088 | 0 |
| TNK2 | cg20384732 | 0.0961345 | 0.0640829 | | 0.0853133 | | | 0.151794 | | 0.0849958 | 8.49555 | 0.0961345 | 0.0113159 | 0 |
| MAPRE3 | cg07495389 | 0.0958001 | 0.111427 | | 0.0425274 | | | 0.0607893 | | 0.151663 | 4.26491 | 0.0958001 | 0.0224624 | 0.00675692 |
| ABLIM1 | cg03850986 | 0.0955978 | 0.0842021 | | 0.0722181 | | | 0.061608 | | 0.169845 | 4.39472 | 0.0955978 | 0.0217529 | 0 |
| ARF4 | cg02491003 | 0.0951964 | 0.118489 | | 0.0390173 | | | 0.0376072 | | 0.16869 | 3.93234 | 0.0951964 | 0.0242086 | 0.0204183 |
| BAHCC1 | cg10294853 | 0.095088 | 0.0579752 | | 0.0767043 | | | 0.160782 | | 0.0847925 | 6.23951 | 0.095088 | 0.0152397 | 0 |
| FOSL2 | cg08836542 | 0.0947478 | 0.101727 | | 0.0525883 | | | 0.0628352 | | 0.152086 | 4.3494 | 0.0947478 | 0.0217841 | 0 |
| TMEM91 | cg12955084 | 0.0947167 | 0.0757481 | | 0.0550433 | | | 0.0918468 | | 0.154385 | 6.52134 | 0.0947167 | 0.0145241 | 0 |
| ADAMTS9 | cg07777540 | 0.0945958 | 0.0568261 | | 0.106198 | | | 0.0704567 | | 0.172096 | 4.6656 | 0.0945958 | 0.0202751 | 0 |
| IRS2 | cg24526103 | 0.0945895 | 0.0812619 | | 0.07028 | | | 0.0788823 | | 0.150446 | 4.01723 | 0.0945895 | 0.023546 | 0.0204183 |
| AURKC | cg25802888 | 0.0943435 | 0.0616468 | | 0.0790984 | | | 0.169262 | | 0.0641578 | 8.58275 | 0.0943435 | 0.0109922 | 0 |
| EPAS1 | cg19311375 | 0.0936339 | 0.0722845 | | 0.0936894 | | | 0.0541911 | | 0.17295 | 3.83993 | 0.0936339 | 0.0243843 | 0.0204183 |
| TBC1D22A | cg13223858 | 0.0934808 | 0.0777936 | | 0.079903 | | | 0.146608 | | 0.0627639 | 8.04244 | 0.0934808 | 0.0116234 | 0 |
| NAT14 | cg06423357 | 0.0926348 | 0.0674943 | | 0.105592 | | | 0.167805 | | 0.0310712 | 6.57135 | 0.0988587 | 0.0150439 | 0 |
| MAD1L1 | cg04396112 | 0.0926015 | 0.0665482 | | 0.06692 | | | 0.159509 | | 0.06937 | 8.09623 | 0.0926015 | 0.0114376 | 0 |
| BAT2 | cg05182583 | 0.0925936 | 0.0647622 | | 0.0687353 | | | 0.151571 | | 0.080269 | 7.67491 | 0.0873473 | 0.0113809 | 0 |
| NXN | cg25364619 | 0.0925606 | 0.0730337 | | 0.0326334 | | | 0.145945 | | 0.0997391 | 4.46512 | 0.0922775 | 0.0206663 | 0 |
| THRA | cg01341751 | 0.092157 | 0.0546083 | | 0.0752887 | | | 0.15892 | | 0.0801717 | 5.95922 | 0.0935976 | 0.0157064 | 0 |
| SPATA12 | cg13745870 | 0.0920574 | 0.100235 | | 0.0653707 | | | 0.0378168 | | 0.16356 | 3.99242 | 0.0920574 | 0.023058 | 0.0204183 |
| LOC284009 | cg07013325 | 0.0919667 | 0.0591383 | | 0.0515106 | | | 0.109762 | | 0.148175 | 5.56271 | 0.0919667 | 0.0165327 | 0 |
| SNX29 | cg05203809 | 0.0919461 | 0.0840667 | | 0.101677 | | | 0.0388125 | | 0.160714 | 4.2568 | 0.0919461 | 0.0215998 | 0.00675692 |
| SLC38A8 | cg08382534 | 0.0919411 | 0.0820753 | | 0.0491086 | | | 0.0839862 | | 0.146268 | 4.81172 | 0.0919411 | 0.0191077 | 0 |
| SDC1 | cg22344841 | 0.0915985 | 0.0800003 | | 0.0325684 | | | 0.0943528 | | 0.147012 | 4.63101 | 0.0915985 | 0.0197794 | 0 |
| SGMS1 | cg24004665 | 0.0915425 | 0.0578693 | | 0.175026 | | | 0.0532647 | | 0.129547 | 4.47731 | 0.0915425 | 0.0204459 | 0 |
| WWOX | cg02748089 | 0.0914923 | 0.0641465 | | 0.0454631 | | | 0.151171 | | 0.0931173 | 4.74245 | 0.0914923 | 0.0192922 | 0 |
| KLF6 | cg23680451 | 0.0911624 | 0.0378128 | | 0.154042 | | | 0.0972918 | | 0.119816 | 4.60389 | 0.0911624 | 0.0198012 | 0 |
| NMT1 | cg24136288 | 0.091001 | 0.0629033 | | 0.0891164 | | | 0.149874 | | 0.0638192 | 7.3036 | 0.091001 | 0.0124597 | 0 |
| NFIL3 | cg14290576 | 0.0906716 | 0.0966102 | | 0.0964127 | | | 0.0273118 | | 0.153777 | 3.861 | 0.0906716 | 0.0234839 | 0.0204183 |
| GAS7 | cg13773741 | 0.0905838 | 0.0904777 | | 0.0848183 | | | 0.0407713 | | 0.154554 | 4.51606 | 0.0905838 | 0.0200582 | 0 |
| GEFT | cg15409097 | 0.090561 | 0.0736022 | | 0.0585713 | | | 0.149916 | | 0.067166 | 5.35185 | 0.090561 | 0.0169214 | 0 |
| NEDD9 | cg25250968 | 0.0905219 | 0.0753413 | | 0.148357 | | | 0.0688113 | | 0.0988613 | 5.50152 | 0.0905219 | 0.016454 | 0 |
| VCAN | cg26496628 | 0.0903259 | 0.0806266 | | 0.109863 | | | 0.0447063 | | 0.145942 | 4.4608 | 0.0903259 | 0.0202488 | 0 |
| RAB11FIP1 | cg10211776 | 0.0902803 | 0.103409 | | 0.0337279 | | | 0.0314767 | | 0.180738 | 4.06116 | 0.0902803 | 0.0222301 | 0.00675692 |
| TMEM189 | cg22941573 | 0.0893045 | 0.0862601 | | 0.0396164 | | | 0.0554151 | | 0.16932 | 3.94587 | 0.0893045 | 0.0226324 | 0.0204183 |
| SLC4A8 | cg18077304 | 0.0892005 | 0.0754241 | | 0.0831876 | | | 0.0543929 | | 0.155843 | 4.40656 | 0.0892005 | 0.0202427 | 0 |
| RASAL2 | cg14515791 | 0.0889006 | 0.0551278 | | 0.0775986 | | | 0.0713787 | | 0.168498 | 3.84462 | 0.0889006 | 0.0231234 | 0.0204183 |
| BAG3 | cg17076667 | 0.0888719 | 0.104816 | | 0.0334837 | | | 0.043639 | | 0.158008 | 3.93148 | 0.0891559 | 0.0226774 | 0.0204183 |
| MIR548F5 | cg12029639 | 0.088473 | 0.0646294 | | 0.0782004 | | | 0.151427 | | 0.055884 | 5.09689 | 0.088473 | 0.0173582 | 0 |
| DUSP5 | cg10080966 | 0.0877189 | 0.0924901 | | 0.0466704 | | | 0.036792 | | 0.170408 | 4.13516 | 0.0877189 | 0.021213 | 0.00675692 |
| DGKD | cg08436419 | 0.0876075 | 0.0746357 | | 0.0429249 | | | 0.148179 | | 0.0656574 | 4.27905 | 0.0876075 | 0.0204736 | 0.00675692 |
| SPG20 | cg25179758 | 0.0876068 | 0.0690481 | | 0.0742333 | | | 0.0589413 | | 0.159205 | 5.25769 | 0.0876068 | 0.0166626 | 0 |
| TCF12 | cg24079727 | 0.0871194 | 0.100589 | | 0.0261743 | | | 0.0554953 | | 0.147525 | 5.26395 | 0.0871194 | 0.0165502 | 0 |
| PNPLA2 | cg24427660 | 0.0854743 | 0.0924977 | | 0.0467929 | | | 0.0248275 | | 0.174792 | 3.76108 | 0.085442 | 0.0227174 | 0.0204183 |
| CD109 | cg01606773 | 0.0852308 | 0.0732377 | | 0.0348869 | | | 0.0733369 | | 0.152734 | 4.22607 | 0.0852308 | 0.0201678 | 0.00675692 |
| SELPLG | cg00159243 | 0.0848491 | 0.0411785 | | 0.0632001 | | | 0.146845 | | 0.0911138 | 6.3386 | 0.0848491 | 0.0133861 | 0 |
| FAM160B2 | cg06044105 | 0.0836807 | 0.0564091 | | 0.114562 | | | 0.0348286 | | 0.161594 | 4.15943 | 0.0836807 | 0.0201183 | 0.00675692 |
| CDCP2 | cg10965749 | 0.0831898 | 0.0763079 | | 0.0609677 | | | 0.0426953 | | 0.157662 | 3.79162 | 0.0831898 | 0.0219404 | 0.0204183 |
| ZHX1 | cg00078299 | 0.0830516 | 0.0808487 | | 0.149548 | | | 0.0565436 | | 0.0716181 | 4.77711 | 0.0830516 | 0.0173853 | 0 |
| NOD2 | cg26954174 | 0.0828092 | 0.0757157 | | 0.0775484 | | | 0.0407892 | | 0.147556 | 4.93641 | 0.0828092 | 0.0167752 | 0 |
| OR51A2 | cg25747935 | 0.0825835 | 0.0733783 | | 0.0552507 | | | 0.03716 | | 0.170033 | 4.19135 | 0.0825835 | 0.0197033 | 0.00675692 |
| ANKRD11 | cg26481727 | 0.0825427 | 0.0794393 | | 0.0249966 | | | 0.0548188 | | 0.160749 | 3.93855 | 0.0825427 | 0.0209577 | 0.0204183 |
| PIK3CG | cg24232378 | 0.082496 | 0.0558413 | | 0.0753403 | | | 0.0620272 | | 0.15205 | 4.53039 | 0.082496 | 0.0182095 | 0 |
| GREB1 | cg25649765 | 0.082099 | 0.0909803 | | 0.0738393 | | | 0.0105762 | | 0.160386 | 4.86369 | 0.082099 | 0.01688 | 0 |
| NAALADL2 | cg09890077 | 0.0818195 | 0.091426 | | 0.0889587 | | | 0.0063805 | | 0.152939 | 4.31431 | 0.0818195 | 0.0189647 | 0.00675692 |
| CTSZ | cg16179125 | 0.0764636 | 0.071761 | | 0.149643 | | | 0.0709343 | | 0.0389266 | 6.29268 | 0.0852605 | 0.0135491 | 0 |
|  | cg09400037 | 0.17505 | 0.129888 | | 0.181863 | | | 0.180721 | | 0.23122 | 5.74152 | 0.17505 | 0.0304885 | 0 |
|  | cg12165551 | 0.135269 | 0.125056 | | 0.132007 | | | 0.114713 | | 0.177539 | 5.43612 | 0.135269 | 0.0248834 | 0 |
|  | cg10904856 | 0.133791 | 0.115815 | | 0.144894 | | | 0.15805 | | 0.123872 | 9.61879 | 0.133791 | 0.0139093 | 0 |
|  | cg09354692 | 0.129645 | 0.120995 | | 0.104189 | | | 0.114726 | | 0.178339 | 6.66119 | 0.129645 | 0.0194627 | 0 |
|  | cg20223392 | 0.128868 | 0.13842 | | 0.108061 | | | 0.110457 | | 0.151198 | 5.93979 | 0.128868 | 0.0216957 | 0 |
|  | cg06263395 | 0.128519 | 0.10039 | | 0.122407 | | | 0.178956 | | 0.114465 | 10.4812 | 0.120188 | 0.011467 | 0 |
|  | cg05373251 | 0.123345 | 0.0759221 | | 0.194696 | | | 0.0749307 | | 0.202633 | 3.91483 | 0.123345 | 0.0315073 | 0.0204183 |
|  | cg16454495 | 0.120356 | 0.0884463 | | 0.184909 | | | 0.158535 | | 0.0772206 | 9.23183 | 0.107567 | 0.0116518 | 0 |
|  | cg24704287 | 0.116344 | 0.11908 | | 0.155886 | | | 0.0802014 | | 0.127934 | 6.20725 | 0.121207 | 0.0195267 | 0 |
|  | cg18237047 | 0.113821 | 0.108398 | | 0.125159 | | | 0.0616078 | | 0.176674 | 3.85555 | 0.113821 | 0.0295212 | 0.0204183 |
|  | cg16349093 | 0.113485 | 0.139128 | | 0.067871 | | | 0.0725274 | | 0.156099 | 4.79573 | 0.113485 | 0.0236637 | 0 |
|  | cg07080224 | 0.112962 | 0.133713 | | 0.0781869 | | | 0.0667306 | | 0.161658 | 4.07536 | 0.112962 | 0.0277184 | 0.00675692 |
|  | cg03303585 | 0.112223 | 0.118487 | | 0.0820491 | | | 0.0788804 | | 0.163959 | 4.60308 | 0.112223 | 0.0243799 | 0 |
|  | cg20598190 | 0.108951 | 0.107121 | | 0.0596999 | | | 0.0613533 | | 0.20329 | 5.05124 | 0.108951 | 0.0215692 | 0 |
|  | cg09501509 | 0.107461 | 0.100909 | | 0.0842427 | | | 0.0727631 | | 0.17518 | 4.39935 | 0.107461 | 0.0244266 | 0 |
|  | cg14500300 | 0.107106 | 0.0854423 | | 0.103345 | | | 0.0921763 | | 0.160151 | 5.09432 | 0.107106 | 0.0210246 | 0 |
|  | cg13052638 | 0.106312 | 0.120346 | | 0.160425 | | | 0.0283235 | | 0.140967 | 4.41895 | 0.106312 | 0.0240581 | 0 |
|  | cg23280506 | 0.102411 | 0.0553688 | | 0.173707 | | | 0.158199 | | 0.056123 | 5.34384 | 0.103216 | 0.0193149 | 0 |
|  | cg04860238 | 0.100699 | 0.061309 | | 0.111566 | | | 0.156274 | | 0.0854862 | 8.84594 | 0.100699 | 0.0113836 | 0 |
|  | cg20899781 | 0.100576 | 0.103798 | | 0.0528119 | | | 0.0585192 | | 0.179644 | 4.47646 | 0.100576 | 0.0224677 | 0 |
|  | cg07493197 | 0.100032 | 0.107196 | | 0.156667 | | | 0.0468527 | | 0.113457 | 4.95855 | 0.100032 | 0.0201737 | 0 |
|  | cg07586235 | 0.0997445 | 0.109724 | | 0.0926506 | | | 0.0449423 | | 0.155503 | 3.86883 | 0.0997445 | 0.0257815 | 0.0204183 |
|  | cg19805943 | 0.0995766 | 0.117795 | | 0.068493 | | | 0.0468424 | | 0.157289 | 4.64132 | 0.0995766 | 0.0214543 | 0 |
|  | cg08372212 | 0.0995079 | 0.122123 | | 0.0675287 | | | 0.0386756 | | 0.16097 | 4.80482 | 0.0995079 | 0.02071 | 0 |
|  | cg16908123 | 0.0978121 | 0.0852237 | | 0.0345741 | | | 0.0921733 | | 0.167728 | 4.05884 | 0.0978121 | 0.0240985 | 0.00675692 |
|  | cg24590093 | 0.0950379 | 0.0585758 | | 0.0605089 | | | 0.163856 | | 0.0913194 | 6.73439 | 0.0950379 | 0.0141123 | 0 |
|  | cg00857907 | 0.0936292 | 0.0821585 | | 0.0786567 | | | 0.167693 | | 0.0324393 | 4.63828 | 0.0936292 | 0.0201862 | 0 |
|  | cg10012393 | 0.0920899 | 0.0481014 | | 0.0869814 | | | 0.100781 | | 0.151219 | 4.48652 | 0.0920899 | 0.0205259 | 0 |
|  | cg04958236 | 0.091496 | 0.118744 | | 0.0796299 | | | 0.0113268 | | 0.155133 | 4.03135 | 0.091496 | 0.0226961 | 0.0204183 |
|  | cg01391548 | 0.0909532 | 0.0689011 | | 0.115532 | | | 0.0543638 | | 0.150733 | 4.46249 | 0.0909532 | 0.0203817 | 0 |
|  | cg09187338 | 0.0909264 | 0.0618766 | | 0.0483786 | | | 0.0992895 | | 0.154249 | 5.14019 | 0.0909264 | 0.0176893 | 0 |
|  | cg07809831 | 0.0904534 | 0.0495086 | | 0.0530797 | | | 0.0926498 | | 0.175397 | 3.80945 | 0.0904534 | 0.0237445 | 0.0204183 |
|  | cg10040530 | 0.0902792 | 0.0518633 | | 0.080263 | | | 0.159324 | | 0.0720604 | 5.28607 | 0.0902792 | 0.0170787 | 0 |
|  | cg03784363 | 0.0899423 | 0.0526153 | | 0.0940006 | | | 0.0710688 | | 0.16574 | 4.85978 | 0.0899423 | 0.0185075 | 0 |
|  | cg10007075 | 0.0898765 | 0.0229195 | | 0.130492 | | | 0.0861455 | | 0.166358 | 5.32467 | 0.097202 | 0.018255 | 0 |
|  | cg03524147 | 0.0895546 | 0.100406 | | 0.0791741 | | | 0.0184057 | | 0.165922 | 4.20756 | 0.0895546 | 0.0212842 | 0.00675692 |
|  | cg18866210 | 0.0890506 | 0.101486 | | 0.0490099 | | | 0.03287 | | 0.165843 | 4.32822 | 0.0890506 | 0.0205744 | 0 |
|  | cg24730612 | 0.0883361 | 0.0826963 | | 0.0585067 | | | 0.0464971 | | 0.167883 | 3.83275 | 0.0883361 | 0.0230477 | 0.0204183 |
|  | cg00206507 | 0.0881146 | 0.0812932 | | 0.0780947 | | | 0.0389431 | | 0.164367 | 4.01855 | 0.0881146 | 0.0219269 | 0.0204183 |
|  | cg18398175 | 0.0874664 | 0.0513797 | | 0.0947081 | | | 0.150218 | | 0.0612258 | 7.34137 | 0.0874664 | 0.0119142 | 0 |
|  | cg26791879 | 0.0856489 | 0.0921628 | | 0.0254727 | | | 0.155046 | | 0.0347254 | 4.35865 | 0.0877178 | 0.020125 | 0 |
|  | cg13725826 | 0.0855478 | 0.0831616 | | 0.130402 | | | 0.00291592 | | 0.156888 | 3.97005 | 0.0855478 | 0.0215483 | 0.0204183 |
|  | cg22153312 | 0.0848536 | 0.0505637 | | 0.05132 | | | 0.154718 | | 0.0759241 | 4.73596 | 0.0882694 | 0.0186381 | 0 |
|  | cg08123444 | 0.0831679 | 0.0830631 | | 0.163609 | | | 0.0752717 | | 0.0364914 | 4.89626 | 0.0831679 | 0.016986 | 0 |
|  | cg19856705 | 0.0815495 | 0.0454075 | | 0.0460763 | | | 0.150805 | | 0.0774869 | 4.72066 | 0.0815495 | 0.017275 | 0 |
| DHRS4L2 | cg13555354 | -0.162579 | -0.180767 | | -0.132051 | | | -0.116434 | | -0.21204 | -7.02512 | -0.162579 | 0.0231425 | 0 |
| SLC38A1 | cg17090968 | -0.126032 | -0.0796071 | | -0.122264 | | | -0.119036 | | -0.206703 | -5.24305 | -0.126032 | 0.024038 | 0 |
| LRRFIP1 | cg15579587 | -0.159929 | -0.0849623 | | -0.169794 | | | -0.210488 | | -0.204803 | -7.63004 | -0.159929 | 0.0209604 | 0 |
| FXYD7 | cg25252585 | -0.16786 | -0.120697 | | -0.201237 | | | -0.187606 | | -0.191545 | -9.11352 | -0.16786 | 0.0184188 | 0 |
| NAGK | cg18991240 | -0.111092 | -0.0964384 | | -0.0678304 | | | -0.0918547 | | -0.186439 | -5.20836 | -0.111092 | 0.0213295 | 0 |
| KCNAB2 | cg03400374 | -0.156359 | -0.109081 | | -0.153818 | | | -0.194637 | | -0.183122 | -8.92643 | -0.156359 | 0.0175164 | 0 |
| ALX4 | cg24008884 | -0.10876 | -0.0549762 | | -0.117272 | | | -0.109723 | | -0.182321 | -4.74717 | -0.10876 | 0.0229105 | 0.0204183 |
| BCL11B | cg14841828 | -0.126716 | -0.0748061 | | -0.0936885 | | | -0.172096 | | -0.173246 | -5.49156 | -0.126716 | 0.0230747 | 0 |
| CUX1 | cg12679308 | -0.0961181 | -0.0253872 | | -0.0693193 | | | -0.143801 | | -0.163754 | -5.39159 | -0.0961181 | 0.0178274 | 0 |
| CHD5 | cg21919596 | -0.0884108 | -0.0842243 | | -0.0529901 | | | -0.0519134 | | -0.163282 | -5.44093 | -0.0884108 | 0.0162492 | 0 |
| CSK | cg13578134 | -0.10949 | -0.0387246 | | -0.144657 | | | -0.132659 | | -0.16322 | -5.41525 | -0.10949 | 0.0202189 | 0 |
| IL17D | cg27234864 | -0.108157 | -0.0696398 | | -0.0980091 | | | -0.117234 | | -0.162145 | -5.68295 | -0.108157 | 0.0190319 | 0 |
| TMEM30B | cg04373359 | -0.101358 | -0.0529683 | | -0.0686664 | | | -0.132467 | | -0.159494 | -7.02435 | -0.101358 | 0.0144295 | 0 |
| HSF4 | cg23414876 | -0.122948 | -0.140113 | | -0.0709194 | | | -0.104036 | | -0.156316 | -6.22974 | -0.122948 | 0.0197357 | 0 |
| IRF8 | cg04599946 | -0.110831 | -0.0818449 | | -0.0705474 | | | -0.13591 | | -0.152415 | -4.92815 | -0.110831 | 0.0224895 | 0.0204183 |
| TSPAN4 | cg13682095 | -0.110018 | -0.084912 | | -0.0875089 | | | -0.122998 | | -0.147856 | -5.86869 | -0.110018 | 0.0187466 | 0 |
| HMGA1 | cg01745499 | -0.141095 | -0.116142 | | -0.151044 | | | -0.163139 | | -0.145105 | -11.9066 | -0.141095 | 0.0118501 | 0 |
| LOC100132111 | cg24425838 | -0.12306 | -0.0707799 | | -0.158124 | | | -0.151797 | | -0.142451 | -9.28127 | -0.12306 | 0.013259 | 0 |
| IRX5 | cg09492451 | -0.13474 | -0.0673858 | | -0.155905 | | | -0.203954 | | -0.137898 | -7.8821 | -0.13474 | 0.0170944 | 0 |
| PITX1 | cg25648267 | -0.138228 | -0.144687 | | -0.162549 | | | -0.117348 | | -0.136572 | -7.85602 | -0.14388 | 0.0183147 | 0 |
| TBX1 | cg04999026 | -0.178281 | -0.165084 | | -0.264797 | | | -0.182112 | | -0.132918 | -5.69711 | -0.176069 | 0.030905 | 0 |
| SSPN | cg18702820 | -0.132966 | -0.0788758 | | -0.144866 | | | -0.194823 | | -0.131541 | -10.3377 | -0.132966 | 0.0128622 | 0 |
| ARHGAP23 | cg15825321 | -0.137405 | -0.125417 | | -0.149371 | | | -0.152683 | | -0.12868 | -10.0271 | -0.137405 | 0.0137034 | 0 |
| C5orf38 | cg10958362 | -0.124833 | -0.110778 | | -0.0597876 | | | -0.180047 | | -0.125188 | -6.50931 | -0.124833 | 0.0191775 | 0 |
| IRX3 | cg01135754 | -0.10744 | -0.0610251 | | -0.0891311 | | | -0.171136 | | -0.113445 | -7.34369 | -0.10744 | 0.0146303 | 0 |
| LOC728392 | cg06462347 | -0.108549 | -0.0686164 | | -0.165177 | | | -0.125663 | | -0.108271 | -9.17661 | -0.108549 | 0.0118289 | 0 |
| PTPN6 | cg04956511 | -0.113649 | -0.086318 | | -0.0974141 | | | -0.165043 | | -0.104335 | -8.31444 | -0.10884 | 0.0130905 | 0 |
| CAMK1D | cg00115178 | -0.110072 | -0.0792347 | | -0.108047 | | | -0.154759 | | -0.104121 | -10.3892 | -0.110072 | 0.0105949 | 0 |
| CPE | cg04728296 | -0.0978027 | -0.0517092 | | -0.152787 | | | -0.135407 | | -0.0833294 | -6.15161 | -0.0978027 | 0.0158987 | 0 |
| TNFRSF18 | cg14886269 | -0.0970195 | -0.0675379 | | -0.0903772 | | | -0.150307 | | -0.0819469 | -7.43141 | -0.0970195 | 0.0130553 | 0 |
| TSHR | cg14373410 | -0.0982943 | -0.0568219 | | -0.0885339 | | | -0.172536 | | -0.0782456 | -6.30871 | -0.0982943 | 0.0155807 | 0 |
| ACOT7 | cg18148314 | -0.0816266 | -0.0380247 | | -0.0521249 | | | -0.158521 | | -0.0754077 | -5.1074 | -0.0816266 | 0.015982 | 0.00675692 |
| EZR | cg22871253 | -0.0861072 | -0.0557317 | | -0.0742076 | | | -0.147508 | | -0.0663189 | -6.68756 | -0.0861072 | 0.0128757 | 0 |
| BATF | cg21048162 | -0.0754717 | -0.0469537 | | -0.0518839 | | | -0.152252 | | -0.0426236 | -5.57816 | -0.0754717 | 0.0135299 | 0 |
|  | cg01195276 | -0.0868078 | -0.048044 | | -0.0307666 | | | -0.159238 | | -0.0972657 | -5.06292 | -0.0868078 | 0.0171458 | 0.00675692 |
|  | cg01180552 | -0.0885687 | -0.0511837 | | -0.0397953 | | | -0.15463 | | -0.099514 | -5.85257 | -0.0885687 | 0.0151333 | 0 |
|  | cg08882038 | -0.0872189 | -0.0544917 | | -0.0198073 | | | -0.155933 | | -0.101041 | -6.19177 | -0.0789805 | 0.0127557 | 0 |
|  | cg00841988 | -0.116108 | -0.0832687 | | -0.110707 | | | -0.152187 | | -0.125853 | -7.90309 | -0.116108 | 0.0146914 | 0 |
|  | cg09275704 | -0.11025 | -0.0744122 | | -0.0622481 | | | -0.165284 | | -0.131566 | -7.77926 | -0.11025 | 0.0141723 | 0 |
|  | cg12378888 | -0.13558 | -0.0948218 | | -0.172312 | | | -0.168206 | | -0.131855 | -7.34181 | -0.13558 | 0.0184669 | 0 |
|  | cg24755163 | -0.120524 | -0.150763 | | -0.0577683 | | | -0.108008 | | -0.134112 | -5.34414 | -0.124966 | 0.0233837 | 0 |
|  | cg09950916 | -0.124545 | -0.0871455 | | -0.131486 | | | -0.15703 | | -0.136805 | -11.0486 | -0.124545 | 0.0112725 | 0 |
|  | cg18244483 | -0.10264 | -0.0561372 | | -0.0489147 | | | -0.155505 | | -0.146562 | -6.1238 | -0.10264 | 0.0167608 | 0 |
|  | cg23385248 | -0.141573 | -0.0871528 | | -0.109828 | | | -0.21773 | | -0.154035 | -6.0261 | -0.136955 | 0.022727 | 0 |
|  | cg21072567 | -0.145239 | -0.107535 | | -0.16313 | | | -0.167949 | | -0.162018 | -13.0109 | -0.149662 | 0.0115028 | 0 |
|  | cg24722950 | -0.169127 | -0.123658 | | -0.18695 | | | -0.208282 | | -0.177871 | -13.1089 | -0.169127 | 0.0129018 | 0 |
|  | cg15494597 | -0.109373 | -0.089503 | | -0.0596854 | | | -0.104591 | | -0.179698 | -5.32015 | -0.109373 | 0.0205583 | 0 |
|  | cg14872952 | -0.142424 | -0.094104 | | -0.185345 | | | -0.146112 | | -0.180436 | -7.5394 | -0.142424 | 0.0188907 | 0 |
|  | cg21213973 | -0.102704 | -0.0679038 | | -0.0210893 | | | -0.11617 | | -0.195876 | -5.2379 | -0.102704 | 0.0196079 | 0 |
|  | cg19520927 | -0.137122 | -0.0740599 | | -0.125738 | | | -0.173374 | | -0.196183 | -6.00943 | -0.137122 | 0.0228179 | 0 |
|  | cg21064916 | -0.151176 | -0.112108 | | -0.154086 | | | -0.158657 | | -0.198763 | -6.15351 | -0.151176 | 0.0245674 | 0 |

SAM, Significance Analysis of Microarrays
