# Supplementary material for: Remodeling of the Methylation Landscape in Breast Cancer Metastasis
Source: PLoS One. 2014 Aug 1;9(8):e103896. doi: 10.1371/journal.pone.0103896 (PMC4118917; doi:10.1371/journal.pone.0103896)
Supplement: Table S3 — List of differentially expressed genes. (DOCX) [file pone.0103896.s006.docx]

Table S3. List of differentially expressed genes

| Probe ID | Gene Symbol | Fold Change | q-value |
| --- | --- | --- | --- |
| 204777_s_at | MAL | 2.18644 | 0.0832717 |
| 219667_s_at | BANK1 | 3.11308 | 0.0866425 |
| 221601_s_at | FAIM3 | 2.17347 | 0.0878276 |
| 221234_s_at | BACH2 | 2.33315 | 0.0898764 |
| 210279_at | GPR18 | 2.88359 | 0.0914407 |
| 204674_at | LRMP | 2.11749 | 0.0920232 |
| 206707_x_at | FAM65B | 2.49528 | 0.0920232 |
| 35974_at | LRMP | 2.60835 | 0 |
| 206398_s_at | CD19 | 2.64486 | 0 |
| 212827_at | IGHM | 3.6343 | 0 |
| 205297_s_at | CD79B | 2.60443 | 0 |
| 220059_at | STAP1 | 3.09252 | 0 |
| 210356_x_at | MS4A1 | 3.62568 | 0 |
| 210643_at | TNFSF11 | 2.65723 | 0 |
| 221969_at | NA | 4.45167 | 0 |
| 204581_at | CD22 | 2.50499 | 0 |
| 215118_s_at | NA | 2.77183 | 0 |
| 207389_at | GP1BA | 2.21083 | 0 |
| 41577_at | PPP1R16B | 2.25004 | 0 |
| 212750_at | PPP1R16B | 2.04566 | 0 |
| 206638_at | HTR2B | 2.19876 | 0 |
| 209995_s_at | TCL1A | 4.39102 | 0 |
| 206126_at | CXCR5 | 2.48947 | 0 |
| 206255_at | BLK | 2.24927 | 0 |
| 217418_x_at | MS4A1 | 3.61129 | 0 |
| 205861_at | SPIB | 2.34041 | 0 |
| 39318_at | TCL1A | 4.18638 | 0 |
| 220068_at | VPREB3 | 3.23318 | 0 |
| 205544_s_at | CR2 | 4.92237 | 0 |
| 205935_at | FOXF1 | 2.14678 | 0 |
| 204606_at | CCL21 | 12.1615 | 0 |
| 202992_at | C7 | 6.71179 | 0 |
| 206070_s_at | EPHA3 | 4.23799 | 0 |
| 220301_at | CCDC102B | 2.28024 | 0 |
| 205828_at | MMP3 | 0.0863229 | 0 |
| 213765_at | MFAP5 | 0.272513 | 0 |
| 214927_at | ITGBL1 | 0.362939 | 0 |
| 201069_at | MMP2 | 0.452175 | 0 |
| 209758_s_at | MFAP5 | 0.269493 | 0 |
| 213764_s_at | MFAP5 | 0.274968 | 0 |
| 204948_s_at | FST | 0.476005 | 0 |
| 209596_at | MXRA5 | 0.490107 | 0 |
| 209351_at | KRT14 | 0.248989 | 0 |
| 205422_s_at | ITGBL1 | 0.373324 | 0 |
| 214456_x_at | NA | 0.360108 | 0 |
| 204455_at | DST | 0.10796 | 0 |
| 203699_s_at | DIO2 | 0.320586 | 0 |
| 208607_s_at | NA | 0.368537 | 0 |
| 206326_at | GRP | 0.175366 | 0 |
| 205648_at | WNT2 | 0.393634 | 0 |
| 202450_s_at | CTSK | 0.489301 | 0 |
| 203434_s_at | MME | 0.305223 | 0 |
| 213994_s_at | SPON1 | 0.39977 | 0 |
| 205666_at | FMO1 | 0.409875 | 0 |
| 204475_at | MMP1 | 0.137368 | 0 |
| 205157_s_at | KRT17 | 0.407585 | 0 |
| 203435_s_at | MME | 0.424491 | 0 |
| 213993_at | SPON1 | 0.472015 | 0 |
| 209436_at | SPON1 | 0.475454 | 0 |
| 203700_s_at | DIO2 | 0.345374 | 0 |
| 205226_at | PDGFRL | 0.469081 | 0 |
| 219087_at | ASPN | 0.310225 | 0 |
| 212236_x_at | KRT17 | 0.465703 | 0 |
| 205959_at | MMP13 | 0.215719 | 0 |
| 201860_s_at | PLAT | 0.494991 | 0 |
| 214043_at | PTPRD | 0.436513 | 0 |
| 201820_at | KRT5 | 0.484699 | 0 |
| 204320_at | COL11A1 | 0.378386 | 0 |
| 37892_at | COL11A1 | 0.3718 | 0 |
| 202363_at | SPOCK1 | 0.46849 | 0 |
| 211737_x_at | PTN | 0.231491 | 0 |
| 220356_at | CORIN | 0.492705 | 0 |
| 209465_x_at | PTN | 0.244718 | 0 |
| 213909_at | LRRC15 | 0.450121 | 0 |
| 219454_at | EGFL6 | 0.442318 | 0 |
| 209466_x_at | PTN | 0.220777 | 0 |
| 205907_s_at | OMD | 0.473411 | 0 |
| 209101_at | CTGF | 0.459958 | 0 |
| 205792_at | WISP2 | 0.433964 | 0 |
| 205908_s_at | OMD | 0.479638 | 0 |
| 218730_s_at | OGN | 0.467008 | 0 |
| 203980_at | FABP4 | 0.330581 | 0 |
| 214038_at | CCL8 | 0.422197 | 0 |
| 209863_s_at | TP63 | 0.289036 | 0 |
| 219140_s_at | RBP4 | 0.402275 | 0 |
| 209555_s_at | CD36 | 0.443357 | 0 |
| 205913_at | PLIN1 | 0.339116 | 0 |
| 205381_at | LRRC17 | 0.491361 | 0 |
| 206488_s_at | CD36 | 0.48266 | 0 |
| 206825_at | OXTR | 0.238783 | 0 |
| 207175_at | ADIPOQ | 0.310514 | 0 |
| 211653_x_at | AKR1C2 | 0.401827 | 0 |
| 209699_x_at | AKR1C2 | 0.359016 | 0 |
| 206211_at | SELE | 0.452209 | 0 |
| 204151_x_at | AKR1C1 | 0.363313 | 0 |
| 206439_at | EPYC | 0.287316 | 0 |
| 205680_at | MMP10 | 0.407781 | 0 |
| 207092_at | LEP | 0.360675 | 0 |
| 212531_at | LCN2 | 0.488624 | 0 |
| 210020_x_at | CALML3 | 0.362975 | 0 |
| 219308_s_at | AK5 | 0.473171 | 0 |
