# Supplementary material for: Remodeling of the Methylation Landscape in Breast Cancer Metastasis
Source: PLoS One. 2014 Aug 1;9(8):e103896. doi: 10.1371/journal.pone.0103896 (PMC4118917; doi:10.1371/journal.pone.0103896)
Supplement: Table S4 — List of differentially methylated and expressed genes. (DOCX) [file pone.0103896.s007.docx]

| Gene Name | Probe ID | Methylation data | | | | | | Expression data | | |
| --- | --- | --- | --- | --- | --- | --- | --- | --- | --- | --- |
|  |  | Delta beta-value | | | | | q-value | Fold change | | q-value |
|  |  | ALL | BASAL | HER 2 | LUM A | LUM B |  |  |  |  |
| COL6A1 | cg11401293 | 0.162634 | 0.124995 | 0.189071 | 0.198814 | 0.157171 | 0 | 0.610439 | 0 | |
| C3orf64 | cg22620797 | 0.125782 | 0.071434 | 0.163685 | 0.178213 | 0.117856 | 0 | 0.697666 | 0 | |
| FNDC3B | cg12972064 | 0.113671 | 0.107778 | 0.093797 | 0.092218 | 0.162166 | 0 | 0.7788 | 0 | |
| HTRA1 | cg06474225 | 0.109712 | 0.137099 | 0.07789 | 0.037385 | 0.177697 | 0.0204183 | 0.653908 | 0 | |
| SRPX2 | cg05911774 | 0.102954 | 0.159932 | 0.110964 | 0.027348 | 0.10261 | 0.0204183 | 0.635845 | 0 | |
| MYOF | cg26581982 | 0.10021 | 0.103641 | 0.070663 | 0.048539 | 0.177752 | 0.0204183 | 0.65329 | 0 | |
| IRS2 | cg24526103 | 0.09459 | 0.081262 | 0.07028 | 0.078882 | 0.150446 | 0.0204183 | 0.798014 | 0 | |
| SSPN | cg18702820 | -0.13297 | -0.07888 | -0.14487 | -0.19482 | -0.13154 | 0 | 0.812811 | 0.089765 | |
