# Supplementary material for: Remodeling of the Methylation Landscape in Breast Cancer Metastasis
Source: PLoS One. 2014 Aug 1;9(8):e103896. doi: 10.1371/journal.pone.0103896 (PMC4118917; doi:10.1371/journal.pone.0103896)
Supplement: Materials and Methods S1 — (DOC) [file pone.0103896.s008.doc]

**SI Materials and Methods**

**Patients and Tumor Samples**

Paired primary tumor and lymph node metastases (n=44; Table S1) for methylation (n=44) and gene expression analysis (n=36) were collected at the time of primary surgery with informed consent and banked at MSKCC in accordance with IRB approval. All samples were independently reviewed by a breast cancer pathologist (D.G.). Tumors were microdissected to obtain >70 % tumor cell content.

**DNA methylation Profiling**

Genomic DNA from patient samples was isolated using the DNeasy Blood and Tissue kit (QIAGEN). Nucleic acid quality was determined with the Agilent 2100 Bioanalyzer. Methylation was analyzed on the Infinium 450K methylation chip by the genome core in accordance with manufacturer’s instructions. Methylation data were extracted using GenomeStudio software (Illumina). Methylation values for each site are expressed as a *β* value, based on following calculation: *β* value = (signal intensity of methylation-detection probe)/(signal intensity of methylation-detection probe + signal intensity of non-methylation detection probe).

**Gene Expression Profiling**

RNA was isolated from fresh frozen samples using the RNAeasy miniprep kit (QIAGEN). Primary tumor and lymph node metastasis pairs for which RNA of sufficient quantity and quality was available (n=36) were analyzed on Affymetrix GeneChip Human Genome U133 2.0 Array at the genome core of the Memorial Sloan-Kettering Institute according to the manufacturer’s protocol.

**Clustering Analysis and Measurement of Differential Gene Expression and DNA Methylation**

All analysis was performed using Partek Genomics Suite (Partek), unless otherwise specified. For unsupervised consensus clustering we used 1,000-24,000 most variable methylation probes across the data set. For subtype-specific methylation analysis, analysis of variance (ANOVA) with false discovery rate correction (FDR) on logit-transformed data was used to identify differentially expressed probes between primary tumors and metastases. To identify differentially methylated probes across all subtypes, paired significance analysis of microarrays (SAM) method was performed using R statistical software (v.2.13.0; [http://www.R-project.org](http://www.r-project.org/)). Probes were considered significantly differentially methylated if they displayed an adjusted p-value of <0.01. To identify differentially expressed genes, SAM was performed using Multiple Experiment Viewer (MeV) hosted at SourceForge (<http://sourceforge.net/projects/mev-tm4/>). Genes were considered significantly differentially expressed between primaries and matched metastases if they displayed a 2-fold change and adjusted p-value of <0.01.

Gene set enrichment analysis was performed using GSEA Preranked v1software as described by Subramanian et al. using web-based GenePattern interface . Gene ontology classification was performed using DAVID ([http://david.abcc.ncifcrf.gov/](http://www.sciencedirect.com/science?_ob=RedirectURL&_method=externObjLink&_locator=url&_cdi=272198&_issn=10972765&_origin=article&_zone=art_page&_plusSign=%2B&_targetURL=http%253A%252F%252Fdavid.abcc.ncifcrf.gov%252F)) Pathway analysis was performed using Ingenuity Pathways Analysis (IPA, Version 9.0; Ingenuity Systems®, [http://www.ingenuity.com](http://www.ingenuity.com/))
