# Supplementary material for: Remodeling of the Methylation Landscape in Breast Cancer Metastasis
Source: PLoS One. 2014 Aug 1;9(8):e103896. doi: 10.1371/journal.pone.0103896 (PMC4118917; doi:10.1371/journal.pone.0103896)
Supplement: Figure S3 — Heterogeneity of methylation change on shared metastasis-specific differentially methylated CpGs by molecular subtype. β-values from primary tumors (blue) and metastases (red) are shown for select probes. (PDF) [file pone.0103896.s003.pdf]

Supplementary Figure 3.

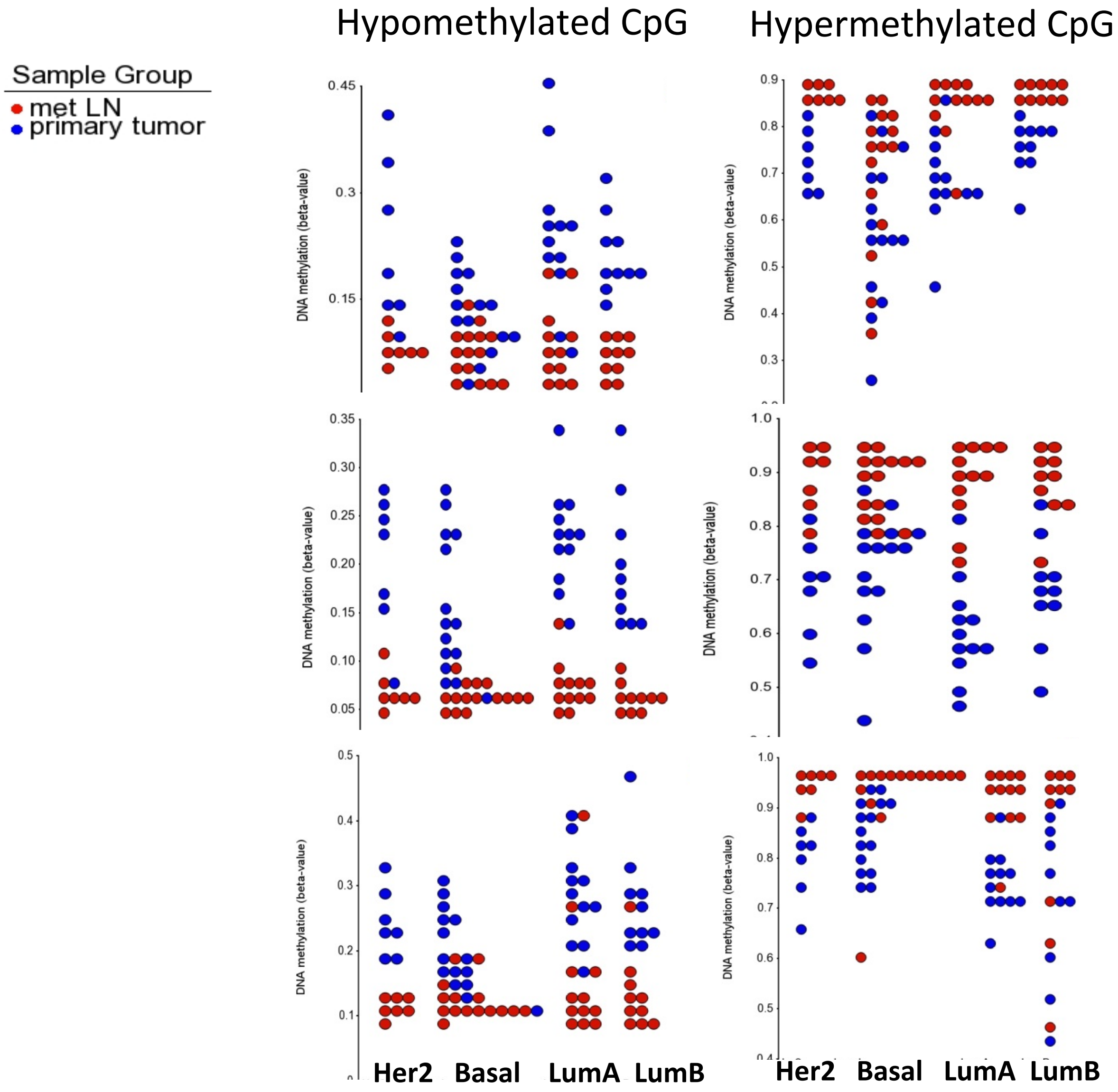

Figure S3. Heterogeneity of methylation change on shared metastasis-specific differentially methylated CpGs by molecular subtype. β-values from primary tumors (blue) and metastases (red) are shown for select probes.
